# A Tumor Microenvironment Model of Pancreatic Cancer to Elucidate Responses toward Immunotherapy

**DOI:** 10.1002/adhm.202201907

**Published:** 2022-12-11

**Authors:** Verena Kast, Ali Nadernezhad, Dagmar Pette, Anastasiia Gabrielyan, Maximilian Fusenig, Kim C. Honselmann, Daniel E. Stange, Carsten Werner, Daniela Loessner

**Affiliations:** ^1^ Leibniz Institute of Polymer Research Dresden e.V Max Bergmann Centre of Biomaterials Hohe Straße 6 01069 Dresden Germany; ^2^ Department of Surgery University Medical Center Schleswig‐Holstein, Campus Lübeck 23562 Lübeck Germany; ^3^ Department of Visceral, Thoracic and Vascular Surgery University Hospital Carl Gustav Carus Medical Faculty Technical University Dresden 01307 Dresden Germany; ^4^ Center for Regenerative Therapies Dresden Technical University Dresden Fetscherstr. 105 01307 Dresden Germany; ^5^ Department of Chemical and Biological Engineering and Department of Materials Science and Engineering Faculty of Engineering Monash University Melbourne VIC 3800 Australia; ^6^ Department of Anatomy and Developmental Biology Biomedicine Discovery Institute Faculty of Medicine Nursing and Health Sciences Monash University Melbourne VIC 3800 Australia

**Keywords:** 3D cancer models, CD11b agonist, extracellular matrix, hydrogels, immunotherapy

## Abstract

Pancreatic cancer is a devastating malignancy with minimal treatment options. Standard‐of‐care therapy, including surgery and chemotherapy, is unsatisfactory, and therapies harnessing the immune system have been unsuccessful in clinical trials. Resistance to therapy and disease progression are mediated by the tumor microenvironment, which contains excessive amounts of extracellular matrix and stromal cells, acting as a barrier to drug delivery. There is a lack of preclinical pancreatic cancer models that reconstruct the extracellular, cellular, and biomechanical elements of tumor tissues to assess responses toward immunotherapy. To address this limitation and explore the effects of immunotherapy in combination with chemotherapy, a multicellular 3D cancer model using a star‐shaped poly(ethylene glycol)–heparin hydrogel matrix is developed. Human pancreatic cancer cells, cancer‐associated fibroblasts, and myeloid cells are grown encapsulated in hydrogels to mimic key components of tumor tissues, and cell responses toward treatment are assessed. Combining the CD11b agonist ADH‐503 with anti‐PD‐1 immunotherapy and chemotherapy leads to a significant reduction in tumor cell viability, proliferation, metabolic activity, immunomodulation, and secretion of immunosuppressive and tumor growth‐promoting cytokines.

## Graphical abstract

The tumor microenvironment of pancreatic cancer is engineered using star poly(ethylene glycol) (PEG)–heparin hydrogels, that are functionalized with integrin‐binding and protease‐responsive peptides to stimulate cell proliferation and migration. The coculture of cancer cells and patient‐derived stromal cells mimics the multicellular composition of tumor tissues and cancer–immune interactions and allows the testing of immunomodulatory treatments in combination with chemotherapy.

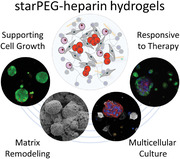

## 1 Introduction

With an overall 5‐year survival rate of around 10%, pancreatic cancer is one of the deadliest malignancies, with a steady increase in worldwide incidence.^[^ ^1^ ^]^ By 2030, it is predicted to be the second highest cause of cancer‐related death.^[^ ^2^ ^]^ In addition to a rising incidence, no improvement in survival has been achieved in decades.^[^ ^2^ ^]^

Pancreatic ductal adenocarcinoma (PDAC), the most common subtype of pancreatic cancer,^[^ ^3^ ^]^ shows an aggressive phenotype,^[^ ^4^ ^]^ intratumoral heterogeneity,^[^ ^5^ ^]^ a characteristic deposition of extracellular matrix (ECM)^[^ ^1^, ^6^ ^]^ and an abundance of stromal cells,^[^ ^5^, ^7^ ^]^ all contributing to resistance to therapy and disease progression.^[^ ^5^, ^8^ ^]^ Within this complex tumor microenvironment (TME), mainly cancer‐associated fibroblasts (CAFs) deposit and remodel the ECM, creating a desmoplastic milieu.^[^ ^1^, ^9^ ^]^ In addition, PDAC has a diverse tumor‐immune landscape.^[^ ^10^ ^]^ Excessive amounts of myeloid cells, including monocytes, granulocytes, and macrophages, invade and populate the tumor tissue.^[^ ^10^, ^11^ ^]^ These cell infiltrates are associated with immunosuppression, T cell exclusion, exhaustion, and dysfunction, resulting in the inability of T cells to attack and destroy the tumor cells.^[^ ^10a^ ^]^

Therapies manipulating mechanisms of immunosuppression to combat tumor growth have shown remarkable success in several malignancies, including melanoma,^[^ ^12^ ^]^ breast,^[^ ^13^ ^]^ and lung cancer.^[^ ^14^ ^]^ Unfortunately, current clinical trials (**Table** **1**) harnessing tumor‐infiltrating immune cells, such as PD‐1 or CTL4‐A immune checkpoint inhibition, have been disappointing in PDAC.^[^ ^15^ ^]^ Hence, there is a strong interest in targeting individual components of the tumor‐immune landscape in PDAC. For example, the migration of immunosuppressive myeloid cells into tumor tissue can be blocked to restore T cell functions.^[^ ^11^, ^16^ ^]^ CD11b/CD18 (*α*M*β*2) is a heterodimeric integrin abundantly expressed on myeloid cells and can be therapeutically manipulated.^[^ ^16^, ^17^ ^]^ CD11b mediates myeloid cell adhesion, trans‐endothelial migration, and cell recruitment into sites of inflammation, negatively regulating proinflammatory pathways.^[^ ^11^, ^17^ ^]^ ADH‐503 (GB1275) is a small‐molecule agonist of CD11b and has shown benefits in pre‐clinical studies in lung^[^ ^16^ ^]^ and pancreatic cancer^[^ ^11a^ ^]^ by suppressing myeloid cell infiltration into sites of inflammation.^[^ ^18^ ^]^ In a PDAC animal model, partial activation of CD11b resulted in the repolarization of immunosuppressive cells into a more proinflammatory phenotype, reduced cell invasion, and an improved T cell response.^[^ ^11a^ ^]^ The combination of ADH‐503 with immunotherapy or standard‐of‐care chemotherapy significantly improved the therapeutic efficiency and survival rates in PDAC animal models,^[^ ^11a^ ^]^ and its clinical relevance was investigated in a first phase I/II clinical trial (NCT04060342, Table 1).

**Table 1:** Selected clinical trials testing the blockade of the interaction between PD‐1 and PDL‐1 in pancreatic cancer (last update October 2022). GEM, gemcitabine; nab‐PTX, paclitaxel

Trial ID	Treatment	Disease	Phase	Status	Refs.
NCT02309177	Nivolumab (PD‐1) + nab‐PTX +/‐ GEM	Locally advanced/metastatic pancreatic cancer	I	Completed	[15a]
NCT02558894	Durvalumab (PDL‐1) +/‐ tremelimumab (CTL4‐A)	Metastatic pancreatic cancer	II	Completed	[15b]
NCT02331251	Pembrolizumab (PD‐1) + nab‐PTX	Metastatic pancreatic cancer	I/II	Completed	[15e]
NCT02879318	nab‐PTX + GEM +/‐ durvalumab (PDL‐1) + tremelimumab (CTL4‐A)	Metastatic pancreatic cancer	II	Active, has results	[15f]
NCT03404960	Niraparib (PARP inhibitor) + nivolumab (PD‐1) or Ipilimumab (CTL4‐A)	Progression‐free pancreatic cancer	I/II	Active	[19]
NCT02866383	Radiotherapy + nivolumab (PD‐1) +/‐ Ipilimumab (CTL4‐A) + after at least one line of prior systemic chemotherapy	Metastatic pancreatic cancer	II	Active	[20]
NCT05052723	Cabozantinib (multi‐target tyrosine kinase inhibitor) + pembrolizumab (PD‐1)	Metastatic pancreatic cancer	II	Recruiting	[21]
NCT02791334	LY3300054 (PDL‐1) + merestinib (multi‐target tyrosine kinase inhibitor)	Advanced, refractory pancreatic cancer	I	Active	[22]
NCT04098432	Radiotherapy followed by nivolumab (PD‐1)	Locally advanced pancreatic cancer	I/II	Active	
NCT04060342	GB1275 (CD11b agonist) +/‐ Pembrolizumab (PD‐1) vs GB1275 + GEM and nab‐PTX	Metastatic pancreatic cancer	I/II	Terminated	
NCT02546531	Defactinib (focal adhesion kinase inhibitor) + pembrolizumab (PD‐1) + GEM	Advanced pancreatic cancer	I	Completed	

Preclinical 3D models of the TME recapitulate the extracellular and cellular components of the immunosuppressive milieu and are beneficial for screening compounds that target specific cell populations of the tumor‐immune landscape.^[^ ^23^ ^]^ However, there is a lack of preclinical PDAC models that can be used for the drug screening of immunotherapeutics. Most immunotherapies are tested in genetically engineered mouse models and patient‐derived xenografts, presenting poor clinical translation because of the lack of human immune components.^[^ ^24^ ^]^ Thus, patient‐derived tumor organoids, established from tumor biopsies or tissue fragments, have been used as an alternative 3D approach.^[^ ^25^ ^]^ They preserve the tissue architecture, cellular complexity, and immune signature of human PDAC.^[^ ^25^ ^]^ Biopsies from pancreatic cancer patients are, however, rare, limiting their application as a high‐throughput platform. Therefore, organoids from primary tumor cells and established cell lines have been generated. However, organoid cultures are typically grown in animal‐derived matrices, such as Matrigel or collagen, suffering from high batch‐to‐batch variation, undefined composition, inherent cellular signaling molecules, and poor mechanical strength.^[^ ^26^ ^]^

To overcome the aforementioned limitations, fully defined synthetic and composite matrices have been developed. These matrices emerged as a powerful bioengineered model to mimic key physiological characteristics of human cancer tissues. For example, self‐assembling peptides coassembled with key ECM molecules present in PDAC tissue have been used to study tumor biology.^[^ ^27^ ^]^ Synthetic hydrogel platforms based on poly(ethylene glycol) (PEG) are popular to mimic the TME.^[^ ^26^, ^28^ ^]^ These synthetic hydrogels offer several advantages over animal‐derived matrices. Not only is the physiological stiffness of the TME observed in patient tumors truly recapitulated, but also the cellular crosstalk by including multiple cell populations.^[^ ^26^, ^28^, ^29^ ^]^ Although these systems have been successfully exploited to model critical characteristics of the pancreatic TME and to study tumor biology, their use as preclinical drug testing platforms for immunotherapeutics has been poorly explored.^[^ ^30^ ^]^

To address this challenge and to model the tumor‐immune cell responses in PDAC, we are using a fully defined and biomimetic 3D model of human PDAC to recreate the stiffness of tumor‐bearing tissue, enable cell‐matrix interactions, and replicate the immunosuppressive TME. Our 3D model is based on matrix metalloproteinase (MMP)‐sensitive four‐arm star‐shaped PEG (starPEG)–heparin hydrogels.^[^ ^26^, ^31^ ^]^ RGD motifs are incorporated to provide cell adhesion sites via integrins, which, combined with the MMP‐responsive motifs, enable cell proliferation and migration. To establish a multicellular TME model, human PDAC cells, together with patient‐derived CAFs and peripheral blood mononuclear cells (PBMCs), are grown encapsulated in different hydrogel matrices. Using our TME model of pancreatic cancer, we aimed to compare cell functions within starPEG–heparin hydrogels and commonly used collagen matrices and to evaluate cell responses toward the novel CD11b agonist ADH‐503 in combination with *α*PD‐1 treatment and standard‐of‐care chemotherapy. By employing our defined TME model of pancreatic cancer for immunotherapeutic strategies, we provide a rationally designed platform to promote the screening of novel therapeutic strategies for patients diagnosed with PDAC.

## 2 Results

### 2.1 Modeling the TME of Pancreatic Cancer Using StarPEG–Heparin Hydrogels

**Figure 1:**
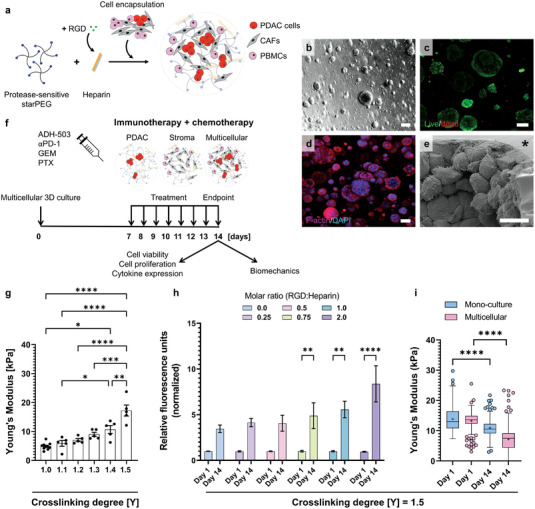
A tumor microenvironment model of pancreatic cancer. a) Schematic illustration of matrix metalloproteinase (MMP)‐sensitive four‐arm star‐shaped poly(ethylene glycol) (starPEG)–heparin hydrogels and cell encapsulation. Hydrogels are formed by crosslinking starPEG with RGD‐functionalized heparin. Pancreatic ductal adenocarcinoma (PDAC) cells, together with cancer‐associated fibroblasts (CAFs) and peripheral blood mononuclear cells (PBMCs), were grown encapsulated in the hydrogel matrices. b) Brightfield microscopy of multicellular 3D cultures on day 14. Scale bar, 200 µm. c) Live (green)/dead (red) staining of multicellular 3D cultures on day 14. Scale bar, 100 µm. d) Fluorescent staining of F‐actin filaments (magenta) and nuclei (blue) of multicellular 3D cultures on day 14. Scale bar, 100 µm. e) Scanning electron microscopy of multicellular 3D cultures on day 14. The asterisk denotes the starPEG–heparin matrix. Scale bar, 10 µm. f) Schematic illustration of the treatment schedule using a combination of immunotherapy and chemotherapy. Multicellular 3D cultures were grown for 7 d before administration of immunotherapy (ADH‐503/*α*PD‐1) and chemotherapy (GEM/PTX), with daily treatment changes for 7 d. On day 14 (endpoint), hydrogels were harvested for downstream analysis. g) Bulk mechanical properties (Young's modulus, kPa) of acellular starPEG–heparin hydrogels on day 1 after polymerization measured by shear rheology. *n* = 3–4, *p ≤ 0.05, **p ≤ 0.01, ***p ≤ 0.001, ****p ≤ 0.001. h) Change in metabolic activity of MIAPaCA‐2 monocultures at a crosslinking degree of 1.5 in response to different RGD concentrations conjugated to heparin measured on day 1 and day 14 of 3D culture. Metabolic activity was normalized to day 1. *n* = 3–4, **p ≤ 0.01, ****p ≤ 0.001. i) Stiffness profile of MIAPaCa‐2 mono‐ and multicellular cultures measured on day 1 and day 14 by AFM to probe the local matrix mechanics (Young's modulus, kPa). *n* = 3, ****p ≤ 0.001.

To model critical parameters of the human pancreatic TME, PDAC cells (BxPC‐3 or MIAPaCa‐2), together with patient‐derived CAFs and PBMCs, were encapsulated within the RGD‐functionalized, protease‐sensitive starPEG–heparin hydrogel matrix (**Figure** **1**a). Cells proliferated homogenously and formed spheroids within 14 d of 3D culture (Figure 1b). On day 14, 3D cultures remained highly viable, indicated by live/dead staining (Figure 1c). The formation of a cytoskeletal network was visualized by F‐actin staining, and nuclei were identified by DAPI staining (Figure 1d). Cells populated the starPEG–heparin matrix and formed densely packed spheroids with the deposition of ECM proteins, as indicated by SEM (Figure 1e).

To assess the suitability of the TME model as a preclinical drug testing platform for the screening of immunotherapeutics and novel combination treatments, PDAC mono‐cultures (BxPC‐3 or MIAPaCa‐2), stromal (CAFs + PBMCs) and multicellular 3D cultures (BxPC‐3 or MIAPaCa‐2 + CAFs + PBMCs) were prepared. 3D cultures were grown for 7 d to allow spheroid formation (Figure 1f). Subsequently, 3D cultures were challenged for additional 7 days with the CD11b agonist ADH‐503 in combination with *α*PD‐1 and chemotherapy (GEM/PTX). We then assessed the cell viability, proliferation, cytokine expression, and biomechanics upon treatment (Figure 1f).

The versatility of the starPEG–heparin hydrogels allowed the precise tuning of the crosslinking density and, therefore, the mechanical properties of the acellular hydrogel matrix. Increasing the ratio of four‐armed starPEG to heparin (*γ*) resulted in hydrogels with different stiffness (Figure 1g). By targeting the upper range for the reported stiffness of PDAC tissue, we selected a crosslinking degree of 1.5 for starPEG–heparin hydrogels throughout our study. To achieve optimal cell adhesion, starPEG–heparin hydrogels were further conjugated with RGD peptides using various concentrations (0‐2 RGD:heparin molar ratio; Figure 1h). The proliferative behavior of encapsulated cells on day 1 and day 14 was closely monitored by light microscopy. At RGD:heparin molar ratios ranging between 0.75 and 2, we observed extensive matrix remodeling due to the cleavage of the matrix metalloproteinase‐responsive sequence and intensive cell outgrowth of the hydrogel. The metabolic activity increased within 14 d by about 5–8 fold (Figure 1h). Based on these findings, we used an RGD:heparin molar ratio of 0.25 to maximize the reproducibility while preventing cell outgrowth. Using this RGD concentration, cells populated the matrix in a more controlled manner, proliferated, and showed a fourfold increase in metabolic activity within 14 d of 3D culture (Figure 1h). The dynamic stiffness remodeling of the starPEG–heparin hydrogel throughout the 3D culture was assessed by indentation using AFM, showing a significant decrease in stiffness of the hydrogel matrix through cleavage of protease‐sensitive crosslinking bonds in both mono‐ and multicellular 3D cultures (Figure 1i).

**Figure 2:**
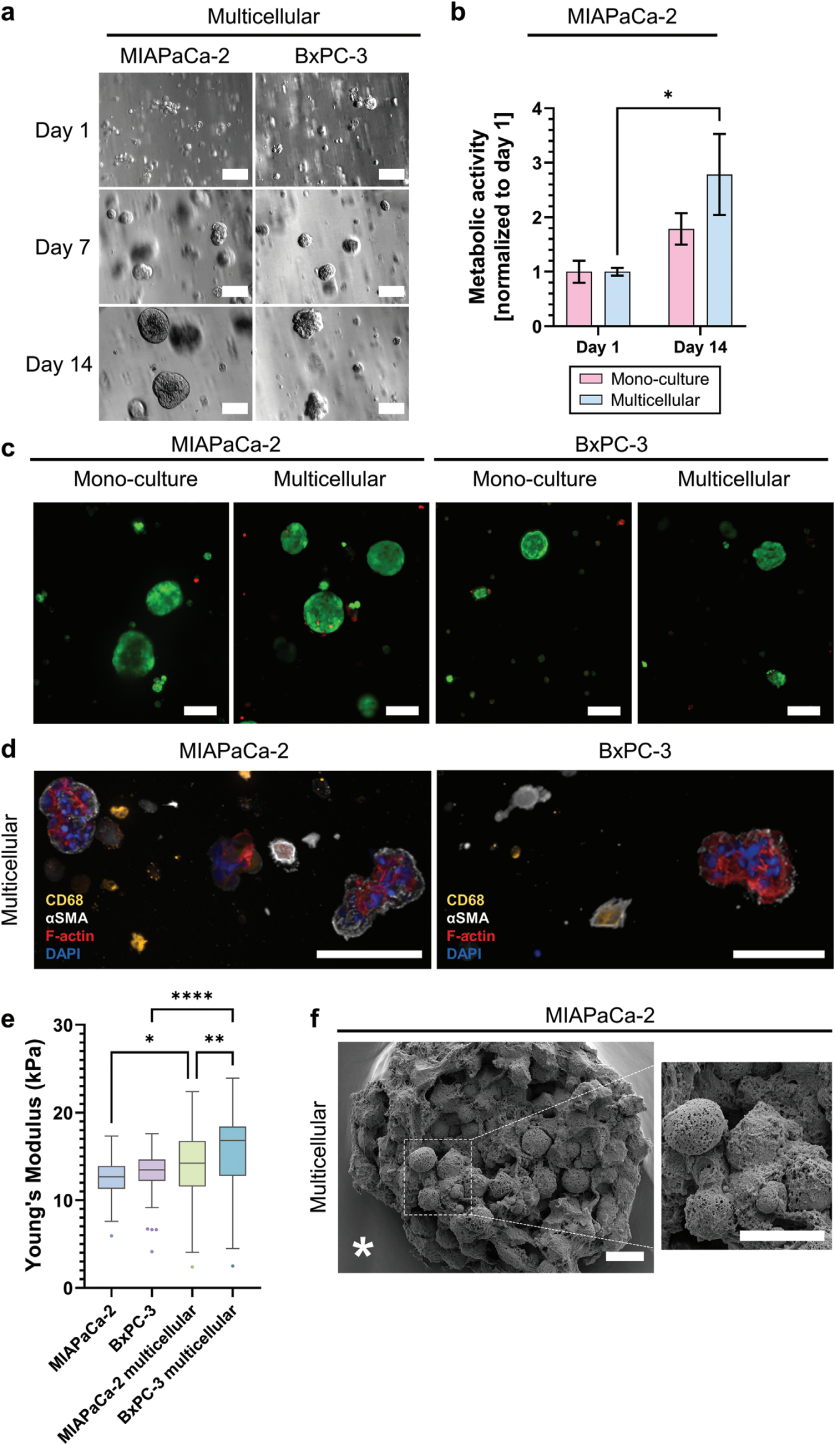
Analysis of the tumor microenvironment model. a) Brightfield microscopy of multicellular 3D cultures showing spheroid formation and growth over 14 d. Scale bar 100 µm. b) Metabolic activity of multicellular 3D cultures significantly increased after 14 d compared to day 1 and cancer cell monocultures. *n* = 3, *p ≤ 0.05. c) Live (green)/dead (red) staining shows that encapsulated cells are highly viable, with multicellular 3D cultures forming more spheroids after 14 d. Scale bar, 100 µm. d) Immunostaining of multicellular 3D cultures confirms the presence of macrophages (CD68, yellow) and cancer‐associated fibroblasts (*α*SMA, white) after 14 d in multicellular 3D cultures, with F‐actin filaments (red) and nuclei (blue) counterstaining. Scale bar, 100 µm. e) Stiffness of cell‐containing starPEG–heparin hydrogels on day 1 of 3D culture. *n* = 3, *p ≤ 0.05, **p ≤ 0.01, ****p ≤ 0.0001. f) Scanning electron microscopy of multicellular 3D cultures on day 14. The asterisk denotes the starPEG–heparin matrix. Scale bar, 10 µm.

We closely monitored the spheroid formation within starPEG–heparin hydrogels by light microscopy on days 1, 7, and 14 (**Figure** **2**a). Multicellular MIAPaCA‐2 cultures formed larger and rounder spheroids compared to BxPC‐3 cultures (Figure 2a). Both mono‐ and multicellular 3D cultures showed increased metabolic activity over 14 days, with multicellular MIAPaCa‐2 cultures being metabolically more active (Figure 2b). These findings were also confirmed by live/dead staining, with multicellular 3D cultures forming more and larger spheroids compared to PDAC monocultures (Figure 2c). Performing laser scanning confocal microscopy, the presence of macrophages was confirmed via CD68 immunostaining, and CAFs were detected via *α*SMA immunostaining (Figure 2d). In multicellular 3D cultures, the cellular composition of spheroids mimicked the composition of tumor tissues, where CAFs surrounded the cancer cells. Differentiated CD68^+^ cells populated the hydrogel matrix, surrounded by spheroids, and were detected within spheroids and in their close proximity (Figure S1, Supporting Information).

### 2.2 Biomechanical Properties of Multicellular 3D Cultures

Tumor‐bearing pancreatic tissue is one of the stiffest among all solid malignancies,^[^ ^32^ ^]^ deregulating mechanosensing pathways to promote disease progression and resistance to therapy.^[^ ^32^, ^33^ ^]^ To model the biomechanics of human PDAC, we tuned the mechanical properties of our hydrogel matrix by increasing the ratio of starPEG to heparin molecules (*γ*), resulting in increased matrix crosslinking. Using this approach, we recapitulated the entire tissue stiffness range observed in PDAC.^[^ ^28^, ^34^ ^]^ Therefore, we used a *γ* of 1.5 and determined the mechanical properties by indentation measurements using AFM (Figure 2e). Biomechanics was tested at the beginning of the 3D culture on day 1 after cell encapsulation in PDAC monocultures (BxPC‐3 or MIAPaCa‐2), stromal (CAFs + PBMCs) and multicellular 3D cultures (BxPC‐3 or MIAPaCa‐2 + CAFs + PBMCs). Both PDAC monocultures and stromal 3D cultures displayed a Young's modulus of 12.7 ± 2.2 kPa, while multicellular BxPC‐3 cultures exhibited a Young's modulus of 15.7 ± 4.7 kPa, and multicellular MIAPaCa‐2 cultures showed a Young's modulus of 14.0 ± 3.9 kPa (Figure 2e). This is in line with the upper tissue stiffness range observed in PDAC patients.^[^ ^28b^ ^]^

Performing SEM, we identified an extensive deposition of ECM in densely packed spheroids within 14 d (Figure 2f). AFM measurements on day 14 showed no significant differences between the stiffness of cell‐containing hydrogel matrices subjected to the different treatments (Figure S2, Supporting Information). Due to the cleavage of the protease‐sensitive crosslinking bonds,^[^ ^26^, ^31^ ^]^ cell‐containing starPEG–heparin hydrogels had a lower Young's modulus on day 14 (Figure S2, Supporting Information) compared to day 1 (Figure 2e). The mechanical characterization results suggest that matrix remodeling dynamics were independent of the treatment. The presence of stromal cell populations in multicellular 3D cultures in different treatment groups did not alter the matrix stiffness and remodeling compared with the PDAC monocultures, which implies the independence of matrix degradation in the different treatment groups.

### 2.3 ADH‐503 Treatment Increased the Sensitivity to Immunotherapy

To exploit our TME model as a screening platform for immunomodulatory treatments, we challenged PDAC mono‐ and multicellular 3D cultures with ADH‐503 in combination with the checkpoint inhibitor Nivolumab (*α*PD‐1). We assessed the responses to ADH‐503/*α*PD‐1 treatment after 7 d by measuring the metabolic activity and DNA content of the cells and compared the results to the control treatment.

**Figure 3:**
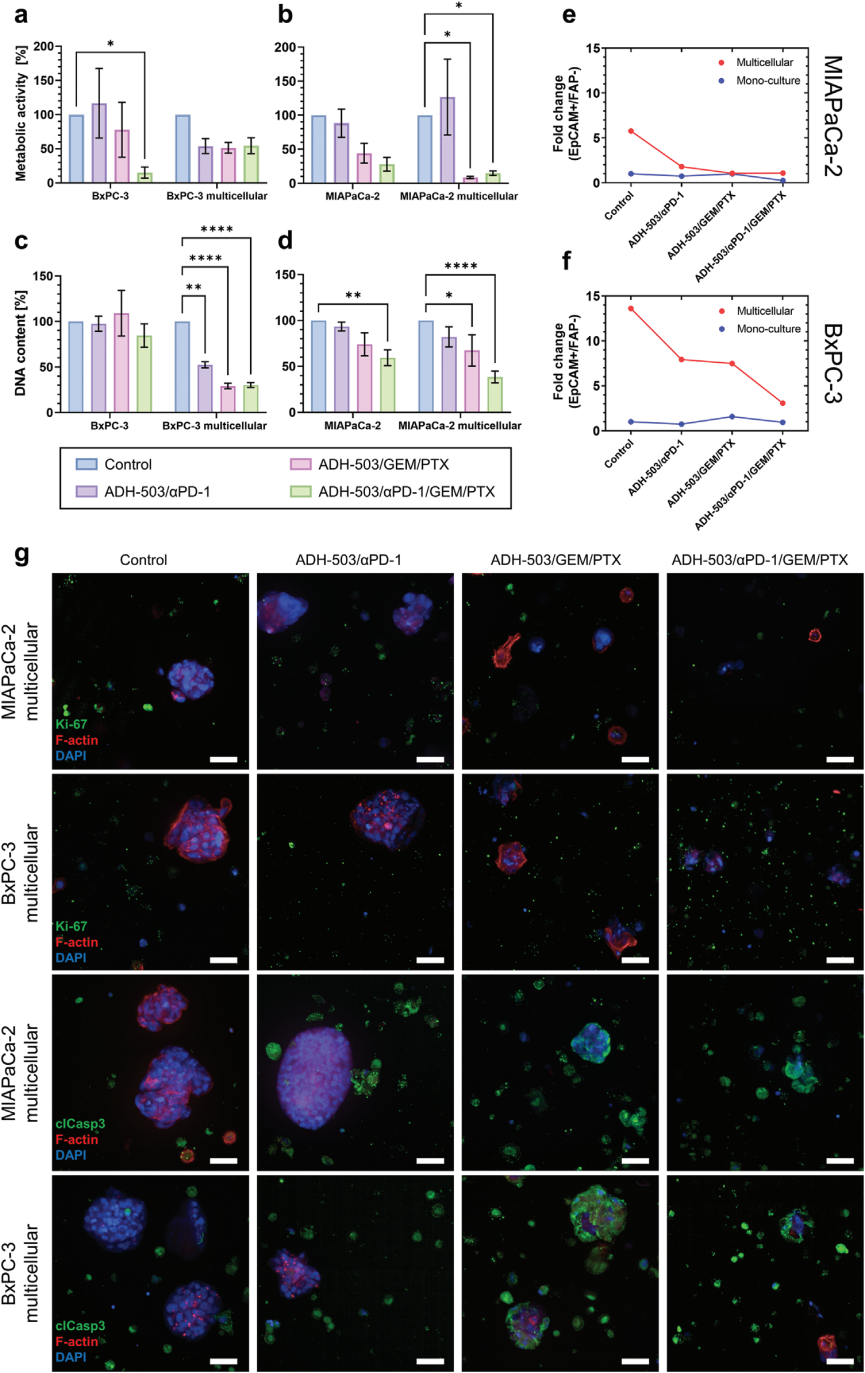
Immunomodulatory treatment attenuates tumor cell functions and improves the efficacy of chemotherapy. Change in metabolic activity of a) BxPC‐3 cultures and b) MIAPaCa‐2 cultures after 7 d of treatment. Change in the DNA content of c) BxPC‐3 cultures, and d) MIAPaCa‐2 cultures after 7 d of treatment. *n* = 3, *p ≤ 0.05, **p ≤ 0.01, ***p ≤ 0.001, ****p ≤ 0.0001. Reduction of e) MIAPaCa‐2, and f) BxPC‐3 cell populations (EpCAM+/FAP‐) in multicellular 3D cultures in response to treatment. Fold change based on the populations normalized to mono‐cultures with control treatment. g) Immunofluorescent staining shows cell proliferation (Ki‐67) and induction of apoptosis (cleaved caspase 3, clCasp3) in multicellular 3D cultures after treatment. Scale bars, 50 µm.

Not surprisingly, PDAC monocultures were almost unresponsive to treatment (**Figure** **3**a–d). In contrast, multicellular 3D cultures showed an increase (MIAPaCa‐2 cells) and a reduction (BxPC‐3 cells) in metabolic activity (Figure 3a,b) and a decrease in their DNA content, with a reduction of the DNA content by 48% for BxPC‐3 cells (Figure 3c) and by 18% for MIAPaCa‐2 cells (Figure 3d). We further investigated these results by flow cytometry (Figure 3e,f). Upon treatment, PDAC cells (EpCAM^+^/FAP^−^) were eradicated in multicellular 3D cultures and mostly unaffected in monocultures (Figure 3e,f). In collagen gels (4 d of treatment), multicellular 3D cultures had a comparable reduction in metabolic activity and DNA content upon treatment (Figure S3, Supporting Information). Stromal cell cultures showed increased metabolic activity (16%) and increased DNA content (11%) upon treatment in starPEG–heparin hydrogels (Figure S4, Supporting Information), which was not observed in the collagen gels (Figure S5, Supporting Information). These findings suggest that our multicellular TME model is suitable for assessing cell responses toward immunomodulatory drug treatment, indicated by a reduction in their DNA content upon treatment.

### 2.4 ADH‐503 Enhanced the Efficacy of Chemotherapy

To assess whether ADH‐503 potentiates the efficacy of chemotherapy, which is a combination of GEM and PTX, we treated PDAC mono‐ and multicellular 3D cultures with ADH‐503 and chemotherapy. Responses to ADH‐503/GEM/PTX treatment were determined after 7 d via the analysis of the cell metabolic activity and DNA content and compared to the control treatment.

BxPC‐3 monocultures were unresponsive to treatment (Figure 3a,c). However, multicellular BxPC‐3 cultures revealed a significant decrease in their DNA content (71%, Figure 3c) and 49% in their metabolic activity (Figure 3a). For MIAPaCa‐2, both mono‐ and multicellular 3D cultures responded to treatment (Figure 3b,d), with monocultures having a reduced metabolic activity (56%, Figure 3b) and DNA content (26%, Figure 3d). Multicellular MIAPaCa‐2 cultures showed a greater response to treatment, with a 92% reduced metabolic activity (Figure 3b) and 33% reduced DNA content (Figure 3d). In collagen gels (4 d of treatment), all PDAC mono‐ and multicellular 3D cultures had significantly reduced metabolic activity (except MIAPaCa‐2 mono‐cultures) and DNA content upon treatment (Figure S3, Supporting Information). Performing flow cytometry, we confirmed that PDAC cells (EpCAM+/FAP‐) in multicellular 3D cultures were more responsive to treatment compared to monocultures (Figure 3e,f, and Figures S6 and S7, Supporting Information). Stromal cell cultures showed again a significantly increased DNA content (15%) upon treatment in starPEG–heparin hydrogels (Figure S4, Supporting Information), which was not observed in the collagen gels (Figure S5, Supporting Information), revealing rather a significantly decreased metabolic activity (40%) and DNA content (36%). Our data suggest that combining ADH‐503 with chemotherapy may improve the cell responses toward cytotoxic therapy.

### 2.5 Combining ADH‐503 with Immunotherapy and Chemotherapy Further Decreased Cell Growth

Combining ADH‐503 with immunotherapy and chemotherapy is a promising approach to potentiate treatment effects further.^[^ ^11a^ ^]^ As such, we treated our 3D cultures using ADH‐503 together with *α*PD‐1 and chemotherapy (PTX/GEM) for 7 d. This treatment combination resulted in the reduction of metabolic activity in almost all conditions (Figure 3a,b). BxPC‐3 monocultures showed a significantly reduced metabolic activity (85%, Figure 3a) but not in the DNA content (Figure 3c). In contrast, multicellular BxPC‐3 cultures revealed a decrease in both metabolic activity (46%, Figure 3a) and DNA content (70%, Figure 3c). MIAPaCa‐2 monocultures had a decreased metabolic activity (72%, Figure 3b) and DNA content (40%, Figure 3d), with multicellular MIAPaCa‐2 cultures benefiting from the treatment with a further reduction in the metabolic activity (85%, Figure 3b) and DNA content (61%, Figure 3d). Performing flow cytometry, we confirmed again that PDAC cells (EpCAM^+^/FAP^−^) in multicellular 3D cultures were more affected by treatment than in mono‐cultures (Figure 3e,f, and Figures S6 and S7, Supporting Information). In collagen gels (4 d of treatment), all PDAC mono‐ and multicellular 3D cultures had a significantly reduced metabolic activity and DNA content upon ADH‐503/*α*PD‐1/GEM/PTX treatment (Figure S3, Supporting Information). Stromal cell cultures were slightly affected by ADH‐503/*α*PD‐1/GEM/PTX treatment in starPEG–heparin hydrogels (Figure S4, Supporting Information), whereas there was again a significant decrease in both metabolic activity (38%) and DNA content (34%) in the collagen gels (Figure S5, Supporting Information).

Performing immunocytochemistry, we identified proliferating cells by the expression of Ki67 in cell‐containing starPEG–heparin hydrogels (Figure 3g), and apoptotic cells were detected by the presence of cleaved caspase 3 (Figure 3g). Overall, we found that both PDAC mono‐ and multicellular 3D cultures were responsive to quadruple treatment. Several drug combinations that also include the CD11b agonist are tested in clinical trials (Table 1), highlighting that CD11 modulation in combination with immunotherapy and chemotherapy may benefit patients diagnosed with PDAC.

### 2.6 Immunomodulatory Drug Treatment Changed the Tumor‐Immune Landscape

Quantifying the cellular composition of encapsulated PBMCs, PDAC patient‐derived PBMCs were compared to PBMCs isolated from a healthy donor using flow cytometry (Table S2, Supporting Information). PDAC patient‐derived PBMCs had a purity of 68% CD45^+^ leukocytes compared to 93% in the healthy donor PBMCs. Both PBMC populations expressed CD11b at comparably high levels (76%). PDAC patient‐derived PBMCs had a higher expression of the immune checkpoint PD‐1 compared to the healthy donor PBMCs. Of note, both types of PBMCs were negative for the macrophage marker CD68 and the epithelial marker EpCAM, showing that CD11b^+^ monocytes were only activated when grown within multicellular 3D cultures.

**Figure 4:**
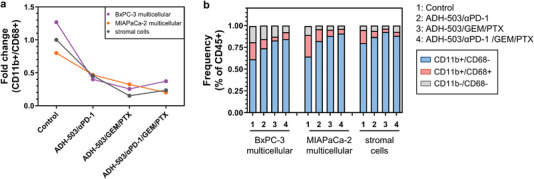
Modulation of the tumor‐immune landscape in response to treatment. a) Change in macrophage population (CD11b^+^/CD68^+^) in multicellular BxPC‐3 and MIAPaCa‐2 cultures, as well as stromal cell cultures after 7 d of treatment. b) Modulation of the total immune cell population (CD45^+^) in multicellular BxPC‐3 and MIAPaCa‐2 cultures, as well as stromal cell cultures after 7 d of treatment.

Having confirmed the presence of CD11b‐expressing macrophages (CD11b^+^/CD68^+^) in our 3D model after 14 d by flow cytometry (**Figure** **4**a,b; Figure S8, Supporting Information), we further explored the potential of ADH‐503 treatment in modulating the tumor‐immune landscape. Compared to the control, we found that ADH‐503/*α*PD‐1 treatment drastically reduced the amount and frequency of CD11b^+^/CD68^+^ cells in the multicellular 3D cultures (Figure 4a,b). This effect was even more pronounced upon ADH‐503/GEM/PTX treatment, and the number of CD11b^+^/CD68^+^ cells slightly increased in response to ADH‐503/*α*PD‐1/GEM/PTX treatment in multicellular BxPC‐3 and stromal cell cultures, but not in the multicellular MIAPaCa‐2 cultures (Figure 4a,b).

We also found an increase in CD11b‐expressing cells in response to all treatments, which did not express the macrophage marker CD68 in multicellular PDAC and stromal cell cultures, referred to as CD11b^+^/CD68^−^ cells (Figure 4b). We also detected the presence of CD11b^−^/CD68^−^ cells, with cell numbers declining in the multicellular PDAC cultures upon treatment and slightly increasing in the stromal cells upon ADH‐503/*α*PD‐1 and ADH‐503/*α*PD‐1/GEM/PTX treatment (Figure 4b). Taken together, we confirmed the presence of CD11^+^/CD68^+^ cells after 14 d of 3D culture. Our results also indicate that immunomodulatory drug treatment modulates the tumor‐immune landscape by reducing the amount and frequency of macrophages and partly increasing the frequency of CD11b^+^ cells, which are not macrophages, within our multicellular 3D model.

### 2.7 Immunomodulatory Drug Treatment Altered the Cytokine Profile

**Figure 5:**
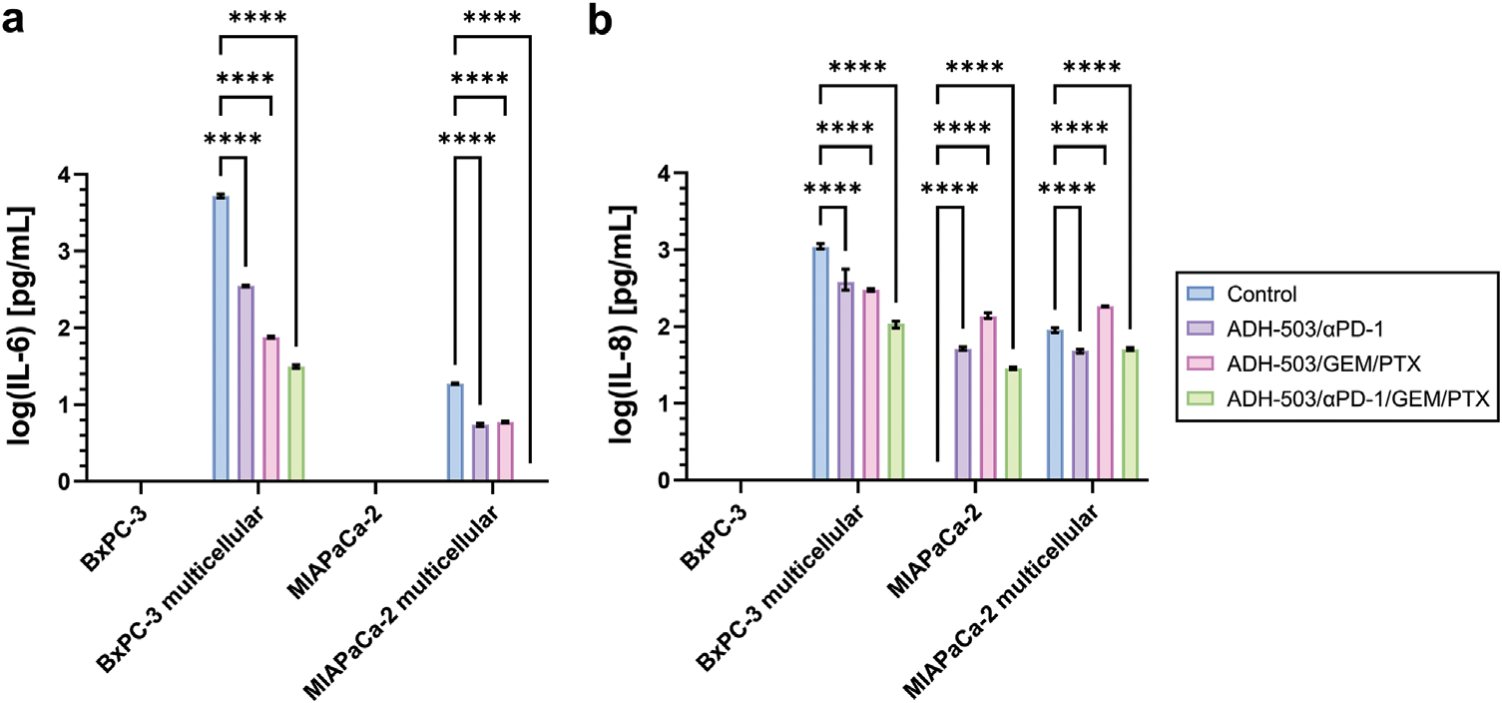
Change in cytokine secretion upon immunomodulatory treatment promotes the generation of a less tumor‐permissive microenvironment. a) Change in IL‐6 secretion in BxPC‐3 and MIAPaCa‐2 mono‐ and multicellular 3D cultures after 7 d of treatment. b) Modification of IL‐8 expression in BxPC‐3 and MIAPaCa‐2 mono‐ and multicellular 3D cultures after 7 days of treatment. *n* = 3, ****p ≤ 0.0001.

Lastly, we asked whether the secretory profile of our TME model is associated with immunosuppression (IL‐6)^[^ ^11^, ^35^ ^]^ and disease progression (IL‐8)^[^ ^36^ ^]^ upon ADH‐503 treatment. While we did not detect IL‐6 (**Figure** **5**a, Figure S9a, Supporting Information) or IL‐8 (Figure 5b, Figure S9b, Supporting Information) in the BxPC‐3 mono‐cultures, we identified robust secretion of IL‐6 (Figure 5a, Figure S9a, Supporting Information) and IL‐8 (Figure 5b, Figure S9b, Supporting Information) in the multicellular BxPC‐3 cultures, with gradually decreasing cytokine levels upon immunomodulatory treatment using starPEG–heparin hydrogels (Figure 5a,b) but heterogeneous expression using collagen gels (Figure S9a,b, Supporting Information). MIAPaCa‐2 mono‐cultures did not secrete IL‐6 (Figure 5a, Figure S9a, Supporting Information), whereas we identified IL‐6 in the multicellular MIAPaCa‐2 cultures (Figure 5a, Figure S9a, Supporting Information). IL‐6 expression gradually decreased upon immunomodulatory treatment and was below the detection limit in response to ADH‐503/*α*PD‐1/GEM/PTX treatment using star‐PEG–heparin hydrogels (Figure 5a) but not using collagen gels (Figure S9a, Supporting Information). IL‐8 secretion was not detected in MIAPaCa‐2 controls using star‐PEG–heparin hydrogels (Figure 5b) but was present using collagen gels (Figure S9b, Supporting Information). The expression of IL‐8 was heterogeneous after immunomodulatory treatment in mono‐ and multicellular 3D cultures using starPEG–heparin hydrogels (Figure 5b) but did not change using collagen gels (Figure S9b, Supporting Information).

Stromal cell cultures did not have any detectable IL‐6 upon treatment using starPEG–heparin hydrogels (Figure S10a, Supporting Information), which was not the case using collagen gels, with significantly reduced IL‐6 levels after treatment (Figure S11a, Supporting Information). In terms of IL‐8, stromal cells grown in starPEG–heparin hydrogels did not have any detectable levels upon ADH‐503/*α*PD‐1 treatment and significantly reduced levels upon GEM/PTX‐containing treatment (Figure S10b, Supporting Information), while in collagen gels, IL‐8 secretion was significantly reduced with all treatments (Figure S11b, Supporting Information). Our results suggest that the presence of stromal cells has a major effect on cytokine secretion. Immunomodulatory treatment led to a reduction of IL‐6 and IL‐8, highlighting the potential of ADH‐503 in affecting the cytokine profile in our TME model.

## 3 Discussion

Human in vitro tissue/disease models based on starPEG–heparin hydrogels are increasingly used to investigate tumor–ECM interactions,^[^ ^37^ ^]^ tumor angiogenesis,^[^ ^26b^ ^]^ or bone metastatic breast cancer.^[^ ^38^ ^]^ This highlights the customization of the material to the desired parameters in terms of physical properties and functionality.^[^ ^31^, ^39^ ^]^ In particular, in vitro models based on starPEG–heparin hydrogels benefit from a defined matrix composition, tunability of mechanical properties, engineered cell binding via tunable RGD conjugation, and cell‐mediated matrix remodeling.^[^ ^26^, ^31^, ^39^ ^]^ In contrast, while 3D approaches based on, for example floating spheroid cultures, allow cells to aggregate and interact with each other, they suffer from the lack of matrix attachment, have limited mechanical strength, show high nutrient and oxygen gradients, and inconsistent cell growth.^[^ ^40^ ^]^

Models of the TME of human PDAC that are applied for drug screening are, however, rare. Thus, hydrogel‐based models of PDAC that replicate the extracellular and cellular complexity of the TME and tumor‐immune landscape are urgently needed to advance translational research and the success of new treatment strategies. Here, we used for the first time a fully defined, multicellular 3D model of human PDAC based on a starPEG–heparin hydrogel system as a preclinical platform to test the response toward novel immunomodulatory strategies in combination with standard‐of‐care chemotherapy.

The dense^[^ ^1b^ ^]^ and immunosuppressive TME of PDAC^[^ ^10^, ^41^ ^]^ have been attributed to unsuccessful clinical trials in which new therapeutics that target tumor‐infiltrating immune cells are tested (Table 1).^[^ ^15a–e^ ^]^ To establish a multicellular TME model, human PDAC cells and patient‐derived CAFs and PBMCs from three different cancer patients were grown encapsulated in a protease‐sensitive starPEG–heparin hydrogel matrix, mimicking the cellular landscape of PDAC. This bioengineered matrix recreated the biomechanical properties, cellular complexity, and immunosuppressive milieu of human PDAC tissues, in which, encapsulated monocytes differentiated into macrophages without external stimulation. Our 3D model supported the viability and growth of multicellular 3D cultures, which were modulated by exposure to immunotherapy and chemotherapy. We demonstrated that combining the CD11b agonist ADH‐503^[^ ^11^, ^16^ ^]^ with *α*PD‐1 immunotherapy and chemotherapy is a promising therapeutic strategy with enhanced efficacy. We showed that immunomodulatory drug treatment changed the tumor‐immune landscape and reduced the expression of immunosuppressive (IL‐6) and tumor growth‐promoting (IL‐8) cytokines. Our TME model allows the pre‐clinical screening of immunotherapeutics and highlights the potential of a novel combination strategy for precision medicine.

Pancreatic cancer is one of the stiffest tumors among all solid malignancies.^[^ ^32^, ^34^ ^]^ Tissue stiffening has been observed as the disease progresses, deregulating mechano‐sensing pathways to promote malignant cell behavior, metastatic spread, and resistance to chemotherapy.^[^ ^32^, ^33^ ^]^ Mimicking the biomechanical characteristics of the diseased matrix is fundamental but remains challenging with current 3D approaches.^[^ ^30^, ^42^ ^]^ To accurately model the biomechanical properties of the diseased matrix, we increased the molar ratio of starPEG to heparin molecules (*γ*), resulting in an increased matrix crosslinking density. This approach allowed us to replicate the entire stiffness range observed in PDAC tissues, which is 5.5 ± 3.2 kPa,^[^ ^34^ ^]^ with 90% of tissues being below 20.1 kPa.^[^ ^28b^ ^]^ Using a *γ* of 1.5, we modeled the biomechanical properties within the upper range of human tumor‐bearing pancreatic tissue, ranging between 10 and 20 kPa.

Mechanical remodeling of the synthetic starPEG–heparin matrix is promoted through the MMP‐mediated cleavage of peptide crosslinkers, which facilitates cell invasion and aggregation.^[^ ^31^, ^39^ ^]^ Consequently, we observed a softening of the matrix on day 14 compared to the start of the 3D cultures. The hydrogel matrices maintained their stiffness within the reported range of PDAC tissue,^[^ ^28b^ ^]^ influencing cell behavior and function throughout the 3D culture and treatment periods.^[^ ^43^ ^]^ The change in stiffness of starPEG–heparin hydrogels was independent of the treatment, suggesting the selectivity of the treatments toward the cell populations rather than having a direct effect on the matrix stiffness.

We tested two representative PDAC cell lines, BxPC‐3 and MIAPaCa‐2 cells, in our 3D model, covering different stages of the disease. BxPC‐3 cells isolated from a patient with no evidence of metastatic spread^[^ ^44^ ^]^ represent an epithelial phenotype with high expression of E‐cadherin.^[^ ^45^ ^]^ MIAPaCa‐2 cells represent a mesenchymal phenotype derived from a patient with detectable tumor infiltrates in the periaortic area^[^ ^44^ ^]^ and have high levels of vimentin.^[^ ^45^ ^]^ Both PDAC cell lines proliferated well when grown encapsulated in starPEG–heparin hydrogels. They formed spheroids within 7 d of 3D culture, with MIAPaCa‐2 cells showing an increased proliferation, spheroid formation, and higher metabolic activity compared to BxPC‐3 cells, which is in line with reported data.^[^ ^44^ ^]^ According to the literature and based on our own experience, BxPC‐3 show a lower cell doubling time of 48–60 h, whereas MIAPaCa‐2 double around every 40 hours.^[^ ^44^ ^]^ This explains the different growth characteristics of both cell lines in our 3D model. Both PDAC cells benefited from the 3D coculture with patient‐derived CAFs and PBMCs, resulting in a dramatic increase in their proliferation, spheroid formation, and metabolic activity, highlighting the key role of the tumor stroma in promoting malignant behavior and disease progression.^[^ ^46^ ^]^ The increase in spheroid growth has been previously described for 3D cocultures^[^ ^47^ ^]^ and is attributed to extensive cellular crosstalk between the different cell types, reflecting the clinical situation more closely than in 3D models that only include one cell population. MIAPaCa‐2 cells escaped the hydrogel matrix when cocultured with stromal cells, indicative of an increased invasive phenotype in the multicellular 3D cultures. SEM imaging revealed that, despite the high stiffness, the starPEG–heparin hydrogel matrix supports cell migration, spheroid formation, and proliferation, by stimulating the deposition of new ECM components. Notably, we found expression of the macrophage marker CD68 after 14 d of 3D culture, highlighting that our TME model recreates tumor‐immune cell interactions and supports the survival of patient‐derived stromal cells. As such, our 3D approach provides a rationally designed platform modulating intra‐tumoral aspects of the tumor‐immune cell responses seen in PDAC.

The success of systemic standard‐of‐care chemotherapy in PDAC patients is disappointingly low.^[^ ^15^, ^48^ ^]^ Cancer cells develop chemoresistance, enabling them to survive and grow.^[^ ^48^ ^]^ In PDAC patients, immunosuppressive myeloid cells populate the tumor area, mediating T cell exclusion, resistance to immune checkpoint inhibition, and poor clinical prognosis.^[^ ^10^, ^11^ ^]^ Targeting myeloid cells by activating CD11b signaling is a promising approach for immune checkpoint therapies. Preclinical data using the CD11b agonist ADH‐503 in animal models have demonstrated benefits in the lung^[^ ^16^ ^]^ and pancreatic cancers.^[^ ^11a^ ^]^ The identification of efficient immunotherapeutics will be tremendously improved by using pre‐clinical models that replicate components of the human tumor‐immune landscape. Therefore, we validated the suitability of our TME model as a drug testing platform using a novel immunotherapeutic and/or combination treatment strategy. We used the CD11b agonist ADH‐503 together with *α*PD‐1 immune checkpoint inhibition and a GEM/PTX combination^[^ ^49^ ^]^ to treat our multicellular 3D cultures. Responses toward treatment were assessed in the two different PDAC cell lines, BxPC‐3 and MIAPaCa‐2, in the presence or absence of stromal cells. Our results highlight that the bioengineered TME model of human PDAC allows for immunomodulatory drug screening, whereas the collagen control matrix led to rather limited cell responses.

Unsurprisingly, PDAC monocultures did not respond to the combination treatment of ADH‐503 and *α*PD‐1. However, when cocultured with patient‐derived CAFs and PBMCs, this immunotherapy slightly reduced the metabolic activity, and significantly decreased the DNA content in multicellular BxPC‐3 cultures, with MIAPaCa‐2 cells remaining unaffected. Clinical trials testing immune checkpoint therapy are primarily evaluated in patients with advanced pancreatic cancer (Table 1), and little is known about the efficacy in early‐stage disease. Besides, various compensatory mechanisms within the cell populations exist, and additional studies using early‐stage PDAC cells will be required to verify our findings. Mechanistically, *α*PD‐1 activation promotes the expansion of CD8 T cells,^[^ ^25^, ^50^ ^]^ and modulation of CD11b induces a change in the immune cell population by reducing the number of immunosuppressive cells while establishing a more proinflammatory TME.^[^ ^11^, ^16^ ^]^ Consistent with our data, ADH‐503 treatment does not affect the viability of tumor or stromal cells.^[^ ^16^ ^]^ Stromal cells showed a slight increase in metabolic activity and DNA content upon immunomodulatory treatment, confirming the effective modulation of the immune cell compartment in response to ADH‐503 treatment in our 3D model. Our findings suggest that ADH‐503 improves *α*PD‐1 therapy, which is in line with previous reports.^[^ ^11a^ ^]^ Although we used patient‐derived PBMCs, we did not confirm the presence of CD8 T cells in our TME model. Further characterization of the present immune effectors is needed to confirm their role in the cell response to ADH‐503 and *α*PD‐1 treatment. To the best of our knowledge, the most recent clinical trial on the efficacy of ADH‐503 as monotherapy or in combination with *α*PD‐1 (NCT04060342) has been recently terminated, as no clear benefit was observed in patients. However, further details on the mechanisms and possible approaches may be identified after the publication of their results.

Preclinical data indicate that ADH‐503 potentiates the efficacy of chemotherapy, reducing tumor growth and disease progression.^[^ ^11a^ ^]^ Chemotherapy in combination with immunotherapy has synergistic effects, enhancing the immunomodulatory response via the upregulation of immune checkpoint ligands.^[^ ^51^ ^]^ GEM, a synthetic pyrimidine nucleoside analog, is incorporated into newly synthesized DNA during cell division, inhibiting DNA synthesis and causing immunomodulatory effects in pancreatic cancer.^[^ ^52^ ^]^ In combination with nab‐PTX, a tubulin‐binding drug that blocks cell cycle progression, prolonged survival has been observed.^[^ ^49^, ^53^ ^]^ Consistent with these reports, our data demonstrate that the combination of ADH‐503 with immune checkpoint inhibition and GEM/PTX resulted in significant tumor control. It has been shown previously^[^ ^54^ ^]^ that both MIAPaCa‐2 and BxPC‐3 cells express PD‐1, which explains the effect of the treatments that include immune checkpoint inhibition. Nevertheless, the treatment efficacy was more pronounced in immunotherapy‐responsive multicellular 3D cultures. Several clinical trials combining immunotherapy with GEM and nab‐PTX are ongoing to determine their efficacy in PDAC (Table 1). Our data underscore the potential of this therapeutic combination approach and warrant further studies.

In contrast to multicellular 3D cultures, PDAC monocultures were less affected by chemotherapy, which is in line with the literature^[^ ^48^ ^]^ and clinical situation,^[^ ^15f^ ^]^ confirming the chemoresistant behavior of cancer cells in our TME model. According to our data, the stromal compartment is less proliferative than cancer cells, making them less affected by cytotoxic treatment. Moreover, the stromal compartment proliferated in response to GEM/PTX combined with immunotherapy since immunotherapy increases, for example, the proliferation of T cells^[^ ^25^ ^]^ and proinflammatory M1 macrophages.^[^ ^11^, ^16^ ^]^

In the pancreatic TME, M1 macrophages are associated with reduced tumor growth, whereas M2 macrophages promote tumor growth and correlate negatively with patient prognosis.^[^ ^10^, ^55^ ^]^ We detected the presence of macrophages (CD68^+^) in our multicellular 3D cultures after 14 d. Whether these macrophages functioned as tumor‐associated macrophages will be addressed in future studies. Furthermore, we observed partial modulation of the tumor‐immune landscape in response to immunomodulatory drug treatment. As indicated in previous reports, CD11b activation results in modulation of the myeloid compartment and reprogramming of macrophages.^[^ ^11a^ ^]^ Specifically, a drastic decrease in M2 macrophages in tumor tissues and a simultaneous increase in the ratio between M1 to M2 macrophages were reported, inducing an antitumor response.^[^ ^11^, ^16^ ^]^ Upon treatment, we found a marked decrease in macrophages, which is in line with previous reports,^[^ ^11^, ^16^ ^]^ and our future analyses will shed light on our 3D model's ratio between M1 and M2 macrophages. Furthermore, we observed an increase in CD45^+^/CD11b^+^ leukocytes, which are not macrophages (CD45^+^/CD11b^+^/CD68^+^) and a decrease in CD11b^−^/CD68^−^ cells in response to treatment. The origin and cellular subtypes of these cell populations will be assessed in future studies.

IL‐6 is a cytokine secreted by cancer and stromal cells, including CAFs and tumor‐associated macrophages,^[^ ^56^ ^]^ and elevated expression has been linked to tumor progression and poor survival in pancreatic cancer patients.^[^ ^56^ ^]^ To assess the potential of ADH‐503 in altering cytokine secretion, we next analyzed the secretory profile in our TME model upon treatment and found significantly reduced IL‐6 levels, which were no longer detectable in multicellular MIAPaCa‐2 cultures in response to ADH‐503/*α*PD‐1/GEM/PTX treatment. In both BxPC‐3 and MIAPaCa‐2 monocultures, cell secretion of IL‐6 was below the detection limit. However, we detected robust IL‐6 levels in multicellular 3D cultures throughout all treatment groups, implicating that IL‐6 is primarily not secreted by cancer cells alone. Stromal cell cultures showed robust IL‐6 expression, which was not detectable in response to immunotherapy with or without chemotherapy. As the stromal compartment was highly viable during treatments, synergistic effects between cancer and stromal cells may result in robust IL‐6 secretion in multicellular 3D cultures, which declined in response to immunomodulatory treatment. Our findings align with previous reports^[^ ^11a^ ^]^ and highlight the formation of a more pro‐inflammatory TME upon immunomodulatory treatment. We further support the potential of CD11b activation in repolarizing immune cells toward a phenotype that may elicit an antitumor response in PDAC.

Next, we screened for IL‐8, a proinflammatory cytokine expressed by cancer and stromal cells,^[^ ^57^ ^]^ promoting disease progression in pancreatic cancer^[^ ^36^ ^]^ and other malignancies.^[^ ^57b^ ^]^ Overall, IL‐8 was below the detection limit in BxPC‐3 monocultures and was present in the multicellular BxPC‐3 cultures, implicating potential synergistic effects and cellular crosstalk between tumor and stromal cells in our TME model. Upon CD11b activation, IL‐8 levels gradually decreased. For MIAPaCa‐2 cells, IL‐8 expression levels were rather heterogeneous and require further studies. However, because of the presence of heparin in our hydrogel system, a glycosaminoglycan with high binding affinities for various biomolecules,^[^ ^58^ ^]^ levels of IL‐6 and IL‐8 might be affected. Further analysis is required to understand how heparin binding impacts cytokine quantification in our 3D model.

Considering the secretion profiles of IL‐6 and IL‐8 in starPEG–heparin hydrogels, we detected mixed results in collagen controls and almost no treatment effect. Using collagen gels for 3D cell cultures has several drawbacks, including weak mechanical properties, undefined composition, and inherent cellular signaling molecules,^[^ ^26^ ^]^ all critical factors influencing malignant progression and immunomodulatory mechanisms in cancer. Thus, we strongly emphasize the use of tailored and bioengineered 3D models, as these platforms precisely mimic key components of tumor‐bearing tissues, which undoubtedly improve drug screening.

## 4 Conclusion

We established a starPEG–heparin hydrogel‐based 3D model of human PDAC that recreates the stiffness of tumor‐bearing tissue, enables cell–matrix interactions, cellular crosstalk, and replicates components of the immunosuppressive TME of the disease. PDAC cells formed tumor spheroids within 7 d of culture and benefited from the 3D co‐culture with patient‐derived CAFs and PBMCs, resulting in an increased spheroid formation, cell proliferation, metabolic activity, and cytokine secretion. We found that the combination of the novel CD11b agonist ADH‐503 with immunotherapy and chemotherapy significantly improved the therapeutic efficiency in our TME model. As such, we provide a novel, rationally designed platform to promote the screening of novel immunomodulatory therapeutic strategies for patients diagnosed with PDAC.

## 5 Experimental Section

### Patient‐Derived Cells and Human PDAC Cells

Human pancreatic CAFs were obtained from three different pancreatic cancer patients from the University Medical Center Schleswig‐Holstein, Campus Lübeck, Department of Surgery, with informed patient consent (Ethics Committee Universität zu Lübeck, approval number 20‐009A). Primary PBMCs isolated from three different pancreatic cancer patients were provided by the University Hospital Carl Gustav Carus, Department of Visceral, Thoracic and Vascular Surgery, Technical University Dresden, and informed consent was obtained from participants prior to cell collection (Ethics Committee Dresden, approval number EK76032013). Human PDAC cells (BxPC‐3, MIAPaCa‐2) were purchased from the Leibniz Institute DSMZ—German Collection of Microorganisms and Cell Cultures. CAFs and PDAC cells were grown in high glucose DMEM + GlutaMAX (Thermo Fisher Scientific, 31966021), supplemented with 10% fetal bovine serum (Sigma‐Aldrich, F7524) and 1% penicillin/streptomycin (Sigma‐Aldrich, P4333). Cells at a maximum density of 70–80% were passaged using TrypLE Express Enzyme (Thermo Fisher Scientific, 12605028) and used within 10 passages. PBMCs were thawed 2 d prior to 3D cell culture and grown in RPMI 1640 (Thermo Fisher Scientific, 11875093), supplemented with 10% fetal bovine serum and 1% penicillin/streptomycin. All cells were grown at 37 °C and 5% CO_2_ under humidified and sterile conditions and confirmed mycoplasma‐free prior to use.

### 3D Cell Culture

Cells were grown encapsulated within protease‐sensitive hydrogels that are based on starPEG covalently crosslinked with heparin, as described previously.^[^ ^26^, ^31^, ^39^ ^]^ Heparin was modified with an average of 8 maleimide groups per molecule of heparin and dissolved in PBS (pH 7.4). Then, 0.25 mol RGD‐SP (H2N‐GCWGGRGDSP‐CONH2, MW 990 Da) per mol of heparin was added to the heparin solution prior to cell encapsulation. PDAC cells (2.5 × 10^5^ cells mL^−1^) were mixed with CAFs (5 × 10^5^ cells mL^−1^) and PBMCs (5 × 10^5^ cells mL^−1^) at a ratio of 1:2:2 in the heparin solution to generate multicellular 3D cultures. PDAC monocultures (2.5 × 10^5^ cells mL^−1^) or stromal cell cultures (CAFs + PBMCs, 5.0 × 10^5^ cells mL^−1^ per cell type) were used as controls. Then, starPEG was dissolved in PBS (pH 3.5) and mixed with the heparin‐cell solution at a ratio of 1:1 to yield a crosslinking ratio (*γ*) of 1.5. Droplets (20 µL) of the cell‐containing hydrogel solution were cast and sandwiched between two sterile, Sigmacote (Sigma Aldrich, SL2) coated glass slides and 1 mm spacers, resulting in hydrogel discs with a diameter of approximately 5 mm. After a 10 min polymerization, hydrogel discs were transferred into 48‐well plates containing culture medium. 3D cell cultures were grown for 14 d and monitored by light microscopy. Collagen gels were used as a control matrix. Therefore, a collagen mixture of 1 mg mL^−1^ was prepared by mixing 1:3 of porcine tendon collagen type‐I (FUJIFILM Wako Chemicals, 631‐00651), 10:10 10× DMEM (Sigma‐Aldrich, D2429), 1:58 1 m sodium hydroxide (Sigma‐Aldrich, S5881), 1:19 sterile water and 1:2 of cells in DMEM. Droplets (100 µL) of the cell‐containing collagen mixture (5.0 × 10^4^ PDAC cells mL^−1^, 1.0 × 10^5^ cells mL^−1^ each for CAFs + PBMCs) were added to 96‐well plates and polymerized at 37 °C for 40 min, before adding culture medium. On the following day, cell‐containing collagen gels were transferred into a 48‐well plate and grown for 5 d.

### Drug Treatment

3D cell cultures using starPEG–heparin hydrogels were grown for 7 d and then treated with ADH‐503 (20 × 10^−6^ m,^[^ ^16^ ^]^ Axon Medchem, 3048) combined with Nivolumab (*α*PD‐1, 10 µg mL^−1^,^[^ ^25^ ^]^ Selleckchem, A2002) and/or gemcitabine (GEM, 100 × 10^−9^ m,^[^ ^42^ ^]^ Sigma‐Aldrich, G6423)/paclitaxel (PTX, 100 × 10^−9^ m,^[^ ^42^ ^]^ Sigma‐Aldrich, T7402) for 7 d, with daily treatment changes considering the mean half‐life of ADH‐503, which is 3.95 to 4.68 h, as measured in different animal models.^[^ ^11a^ ^]^ Human *α*IgG4 (10 µg mL^−1^, BioLegend, 403702) was used as an *α*PD‐1 isotype treatment control. Cell‐containing collagen gels were grown for 1 d and then treated as above for 4 d.

### Analysis of Cell Viability

Cell viability of 3D cell cultures was assessed using a LIVE/DEAD viability/cytotoxicity kit (Thermo Fisher Scientific, L3224) as per manufacturer's instructions. Briefly, live cells were stained using calcein‐AM (green, 2 × 10^−6^ m), and dead cells using ethidium homodimer‐1 (red, 4 × 10^−6^ m). After incubation at 37 °C and 5% CO_2_ for 30 min, hydrogels were washed once with PBS and imaged in phenol red‐free DMEM (Thermo Fisher Scientific) at 37 °C and 5% CO_2_ using a Dragonfly spinning disc confocal microscope (Andor).

### Analysis of Cell Metabolic Activity and Proliferation

Cell metabolic activity and proliferation were assessed as reported previously.^[^ ^28a^ ^]^ Briefly, metabolic activity was determined on day 5 (collagen gels) or day 14 (starPEG–heparin hydrogels) using an AlamarBlue assay (Thermo Fisher Scientific, DAL1025) by incubating 4% AlamarBlue reagent in phenol red‐free DMEM (Thermo Fisher Scientific, 21063029). Cell‐free hydrogels served as negative controls. Fluorescent signals (excitation 544 nm, emission 590 nm) were detected using a fluorescence plate reader (Tecan Spark). Then, hydrogels were washed with PBS and frozen at −80 °C for at least 48 h. Cell proliferation was determined by measuring the DNA content using a CyQuant assay (Thermo Fisher Scientific, C7026). Samples were incubated with proteinase K (0.5 mg mL^−1^, Thermo Fisher Scientific, 25530015) in phosphate‐buffered EDTA at 56 °C overnight and treated with RNase A (1.4 U mL^−1^, Thermo Fisher Scientific, 12091021) at room temperature for 1 h. CyQuant reagent dye was added for 5 min protected from light, and fluorescence signals (excitation 485 nm, emission 520 nm) were detected using a fluorescence plate reader. A *λ*DNA standard curve (0–2 µg mL^−1^) was used to calculate the % change of DNA content per treatment condition and normalized to the controls.

### Immunocytochemistry

3D cell cultures were fixed with 4% paraformaldehyde for 10 min and permeabilized with 0.1% Triton X‐100 (Sigma‐Aldrich, T9284) for 20 min. After washing in PBS, samples were blocked with 2% bovine serum albumin/PBS for 3 h. Primary antibodies (Table S1, Supporting Information) were diluted in blocking solution and incubated at 4 °C for 2 d. After washing in 0.1% bovine serum albumin/PBS, secondary antibodies (Table S1, Supporting Information) and dyes (phalloidin ATTO 633, 0.75 U mL^−1^, ATTO‐TEC, AD 633; DAPI, 0.1 mg mL^−1^, Sigma‐Aldrich, D8417) were diluted in 0.1% bovine serum albumin/PBS and incubated at 4 °C overnight (secondary antibodies) and at room temperature for 1 h (dyes), respectively. Fluorescent images were captured using an Opera Phenix Plus (PerkinElmer).

### Flow Cytometry

For flow cytometry, cells were recovered from hydrogels (4 hydrogels per condition) using collagenase I (1.85 mg mL^−1^, Thermo Fisher Scientific, 17018029) at 37 °C for 1 h with pipetting every now and then. The cell‐containing solution was collected, washed with PBS, incubated in TrypLE Express Enzyme, and centrifuged at 350 × g for 5 min. Samples were incubated with human TruStain FcX Fc receptor blocking solution (BioLegend, 422302) at room temperature for 10 min, followed by fluorescently conjugated antibodies (Table S1, Supporting Information) for 20 min protected from light and washed in cell staining buffer (BioLegend, 420201). Samples were stained with DAPI (0.1 µg mL^−1^) and analyzed using the LSRFortessa flow cytometer (BD Biosciences), equipped with a neutral density filter (ND1.0) to enable the analysis of both PBMCs and autofluorescent PDACs and CAFs, and a high‐throughput sampler for quantitative assessment of hydrogel‐harvested cell populations. Results were plotted with Flowing Software (Turku Bioscience) and FlowJo v10.8.1 (Tree Star Inc). Antibody panels were compensated with compensation particles according to the manufacturer's instructions (BD Biosciences, 552843), and gates were set according to controls of PBMCs, CAFs, MIAPaCa‐2, and BxPC‐3, which were stained with viability dye. Counts of cell populations of interest were normalized to respective stromal (macrophage quantity) or PDAC cells with *α*PD‐1 isotype treatment (EpCAM^+^/FAP^−^ quantity).

### ELISA

Human IL‐6 and IL‐8 ELISAs (R&D Systems, D6050, D8000C) were performed as per manufacturer's instructions using a TECAN Spark plate reader (absorbance 450 nm).

### Scanning Electron Microscopy

Cell morphology was analyzed by scanning electron microscopy (SEM). Cell‐containing hydrogels were fixed in 4% paraformaldehyde at 4 °C for 4 h, followed by serial dehydration in ethanol (30, 50, 70, 90, 100%) for 10 min each. Then, samples were dried using a critical point dryer (CPD 030, BAL‐TEC AG) and sputter coated (SCD 050, BAL‐TEC AG) with a thin layer of gold prior to imaging. Scanning electron micrographs were acquired using a Quattro ESEM in high vacuum mode (Thermo Fisher Scientific).

### Rheology

The bulk shear moduli of starPEG–heparin hydrogels were determined using an ARES LN2 rheometer (TA Instruments, Germany) with 8 mm parallel plate geometry. The storage modulus of hydrogels at 0.2% strain was extracted from oscillatory amplitude sweeps within the shear strain range of 0.01–100% at the frequency of 1 Hz. Each measurement was performed in triplicates. The Young's modulus was calculated from the storage modulus, assuming a Poisson ratio equal to 0.5.

### Atomic Force Microscopy

For the atomic force microscopy (AFM) indentation analysis, a Nanowizard I AFM (JPK Instruments) mounted on an Axiovert 200 inverted microscope (Zeiss) was used, with cell‐containing hydrogels immersed in PBS at room temperature. A polystyrene microsphere with a diameter of 10  µm (Microparticles GmbH) was glued by a two‐component epoxy glue (Araldite) on a tip‐less cantilever (PNP‐TR‐TL, NanoAndNore GmbH) with the nominal spring constant of 0.08 N m^−1^. Before the measurements, a calibration step based on the thermal noise method was performed. Each indentation involved the mapping of a 4 × 4 points array with the spacing of 12.5 µm between each indentation point. The approach velocity was set to 5 µm s^−1^ with a 5 nN contact force. The force–distance curves were fitted using the Herz model for a spherical indenter to obtain the Young's modulus. JPK data processing software (JPK Instruments) was used to analyze the data. For each sample, at least nine mapped areas were collected from three different cross‐sections.

### Statistical Analysis

Data are presented as mean ± standard error for two or three independent experiments with three (AlamarBlue assay, ELISA), four (Flow Cytometry), or six (CyQuant assay) technical replicates using GraphPad Prism 9.4 (GraphPad Software). Numerical data were analyzed using a two‐way Analysis of Variance (ANOVA) with Dunnett's correction for multiple comparisons. AFM results for day 1 are presented as box and whisker plots, with median, first, and third quartiles. Outlier data points were identified using Tukey's test. Numerical data were analyzed using one‐way ANOVA with Tukey's correction for multiple comparisons. AFM data for day 14 are presented as mean, with maximum and minimum values of medians of three independent experiments, and the numerical data was analyzed using two‐way ANOVA with Bonferroni's correction for multiple comparisons. Statistically significant differences are denoted (*p ≤ 0.05; **p ≤ 0.01; ***p  ≤  0.001; ****p ≤ 0.0001).

## Conflict of Interest

The authors declare no conflict of interest.

## Author Contributions

V.K., A.N., and D.L. conceived and designed the experiments. V.K., A.N., D.P., A.G., and M.F. performed the experiments and analyzed the data. K.C.H. and D.E.S. provided the patient‐derived CAFs and PBMCs. D.L. and C.W. supervised the project. V.K., A.N., and D.L. drafted the manuscript. All authors read and revised the manuscript.

## Supporting information

Supporting Information

## Data Availability

The data that support the findings of this study are available on request from the corresponding author. The data are not publicly available due to privacy or ethical restrictions.
