# Supplementary material for: A Tumor Microenvironment Model of Pancreatic Cancer to Elucidate Responses toward Immunotherapy
Source: Adv Healthc Mater. 2022 Dec 11;12(14):2201907. doi: 10.1002/adhm.202201907 (PMC11468239; doi:10.1002/adhm.202201907)
Supplement: Supplementary file 1 — Supporting Information [file ADHM-12-2201907-s001.pdf]

ADVANCED HEALTHCARE MATERIALS

Supporting Information

for *Adv. Healthcare Mater.*, DOI 10.1002/adhm.202201907

A Tumor Microenvironment Model of Pancreatic Cancer to Elucidate Responses toward Immunotherapy

*Verena Kast, Ali Nadernezhad, Dagmar Pette, Anastasiia Gabrielyan, Maximilian Fusenig, Kim C. Honselmann, Daniel E. Stange, Carsten Werner and Daniela Loessner**

Supporting Information

A tumor microenvironment model of pancreatic cancer to elucidate responses toward immunotherapy

Verena Kast[1], *Ali Nadernezhad*[1], *Dagmar Pette*[1], *Anastasiia Gabrielyan*[1], *Maximilian Fusenig*[1], *Kim C Honselmann*[2], *Daniel E Stange*[3], *Carsten Werner*[1,4], *Daniela Loessner*[* 1,5,6]

[1]Leibniz Institute of Polymer Research Dresden e.V., Max Bergmann Centre of Biomaterials, Hohe Straße 6, 01069 Dresden, Germany
[2]Department of Surgery, University Medical Center Schleswig-Holstein, Campus Lübeck, Germany
[3]Department of Visceral, Thoracic and Vascular Surgery, University Hospital Carl Gustav Carus, Medical Faculty, Technical University Dresden, Dresden, Germany
[4]Technical University Dresden, Center for Regenerative Therapies Dresden, Fetscherstr. 105, Dresden, 01307, Germany
[5]Department of Chemical and Biological Engineering and Department of Materials Science and Engineering, Faculty of Engineering, Monash University, Melbourne, VIC 3800, Australia
[6]Department of Anatomy and Developmental Biology, Biomedicine Discovery Institute, Faculty of Medicine, Nursing and Health Sciences, Monash University, Melbourne, VIC 3800, Australia

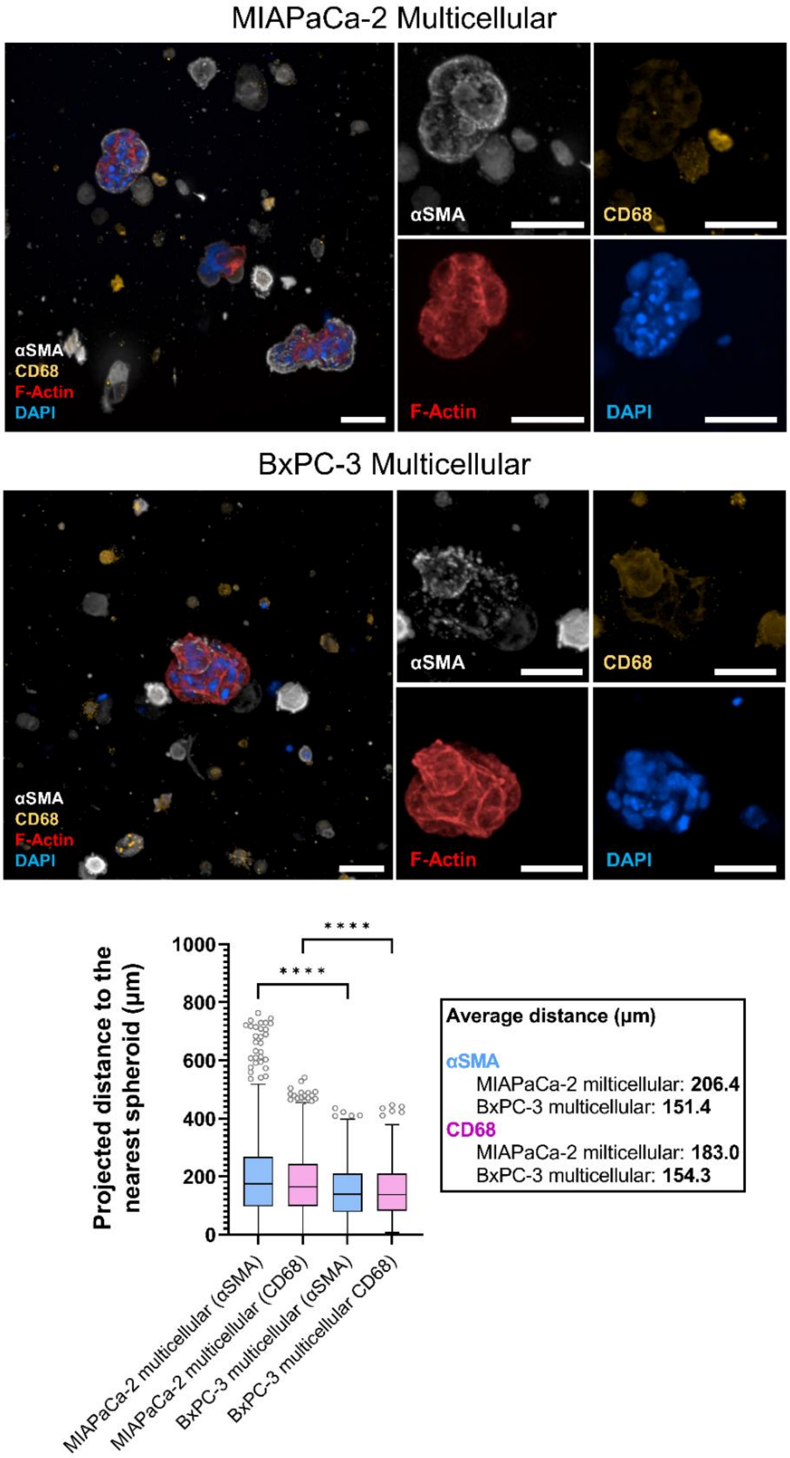

Supplementary Figure S1. Composition of spheroids in multicellular cultures. Immunofluorescence staining shows the presence of different cell types in spheroids in multicellular cultures after 14 days involving MIAPaCa-2 and BxPC-3 cells. Macrophages (CD68, yellow) and cancer-associated fibroblasts (αSMA, white) with F-actin filament (red) and nuclei (blue) counterstaining. Scale bars, 50 μm. The average distance of αSMA+ and CD68+ cells from the nearest spheroids are shown in the lower panel, with open circles denote outliers according to Tukey's analysis.

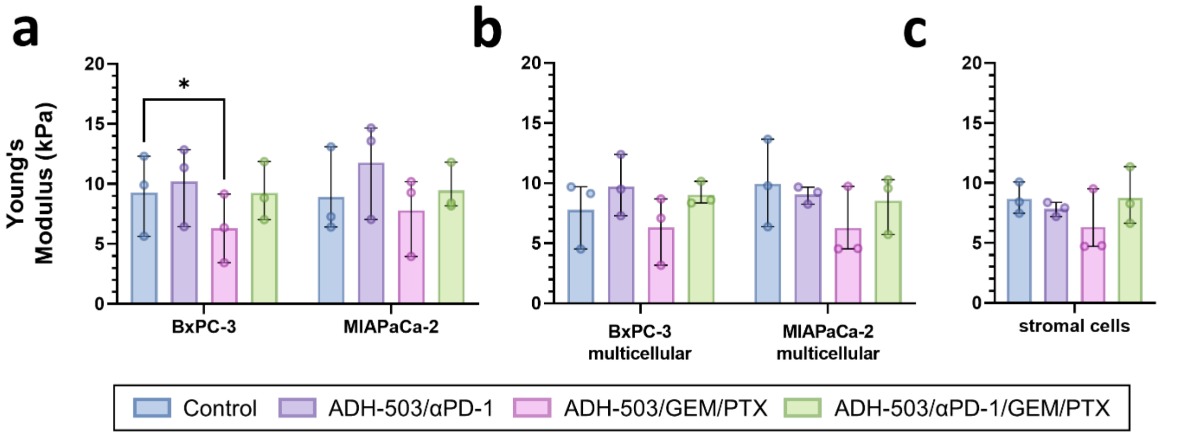

Supplementary Figure S2. Biomechanical properties of the tumor microenvironment model. Stiffness of cell-containing starPEG-heparin hydrogels after 7 days of treatment in 3D cultures in **a,** mono-cultures, **b,** multicellular cultures and **c,** stromal cell cultures. n=3, *$p \leq 0.05$.

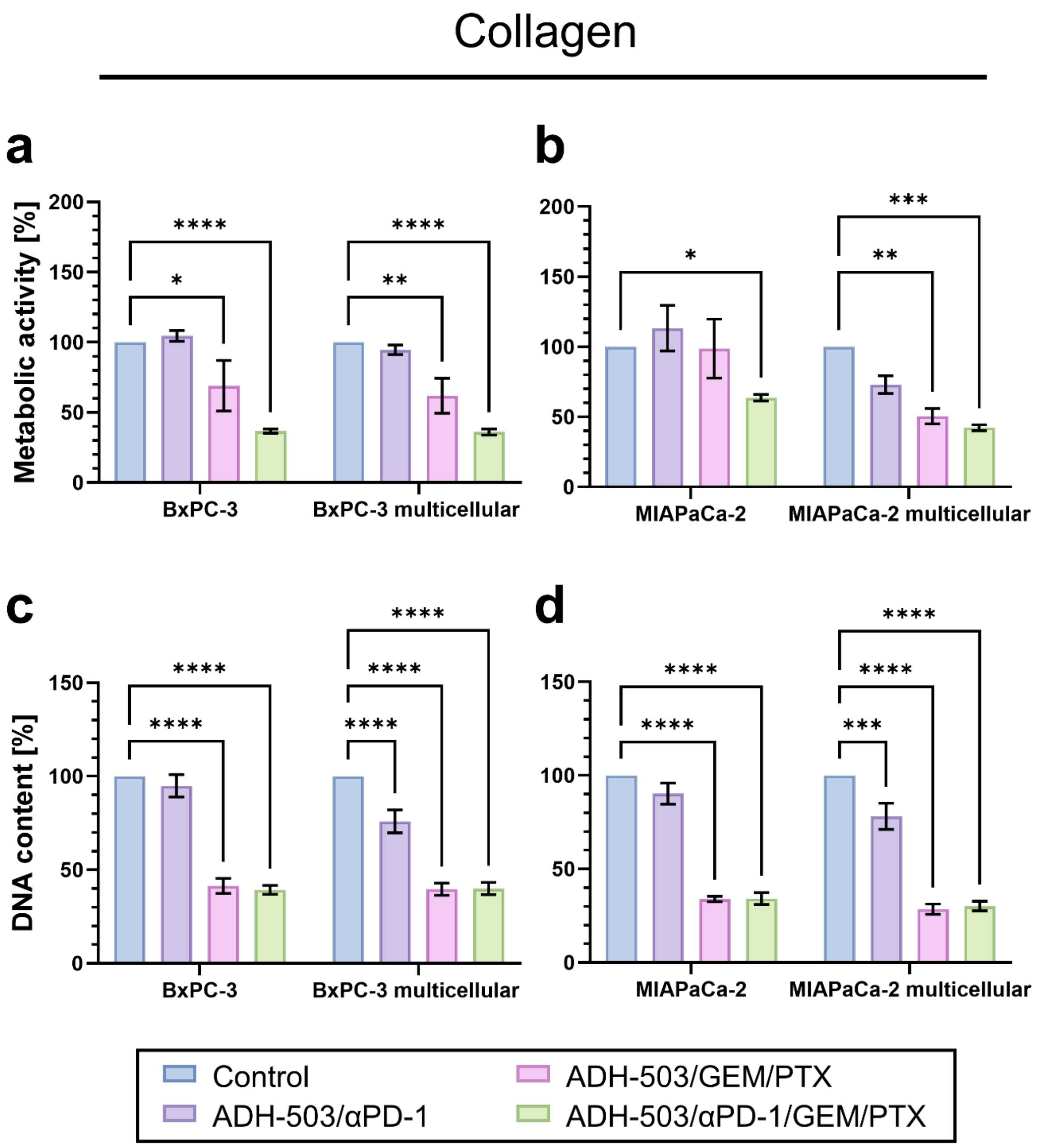

Supplementary Figure S3. Analysis of 3D cell cultures in collagen gels. Change in metabolic activity of **a**, BxPC-3 and **b**, MIAPaCa-2 cultures after 4 days of treatment. Change in the DNA content of **c**, BxPC-3 and **d**, MIAPaCa-2 cultures after 4 days of treatment. n=3, *$p \leq 0.05$, **$p \leq 0.01$, ***$p \leq 0.001$, ****$p \leq 0.0001$.

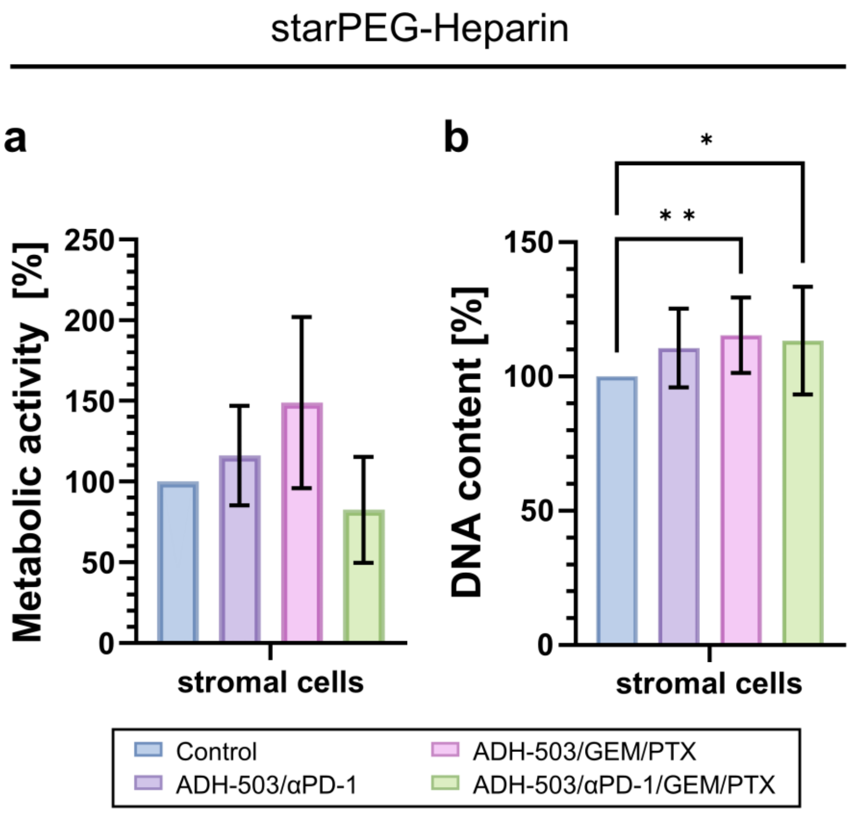

Supplementary Figure S4. Analysis of stromal cell cultures in starPEG-heparin hydrogels. Change in **a**, metabolic activity and **b**, DNA content of stromal cell cultures after 7 days of treatment. n=3, *$p \leq 0.05$, **$p \leq 0.01$.

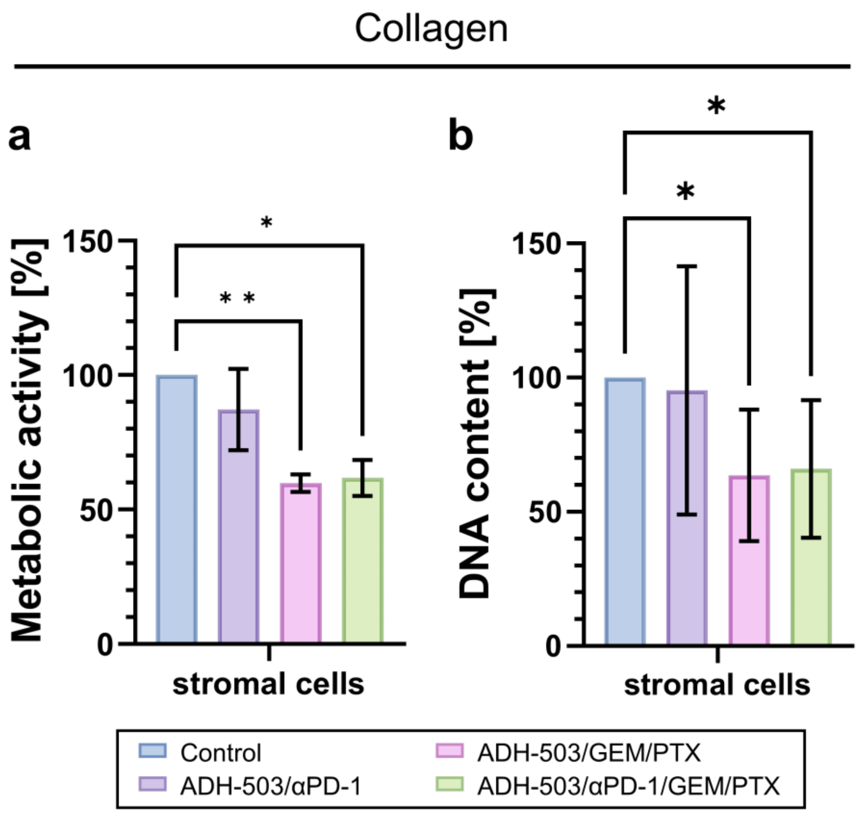

Supplementary Figure S5. Analysis of stromal cell cultures in collagen gels. Change in **a**, metabolic activity and **b**, DNA content of stromal cell cultures after 4 days of treatment. n=3, $*p \leq 0.05$, $**p \leq 0.01$.

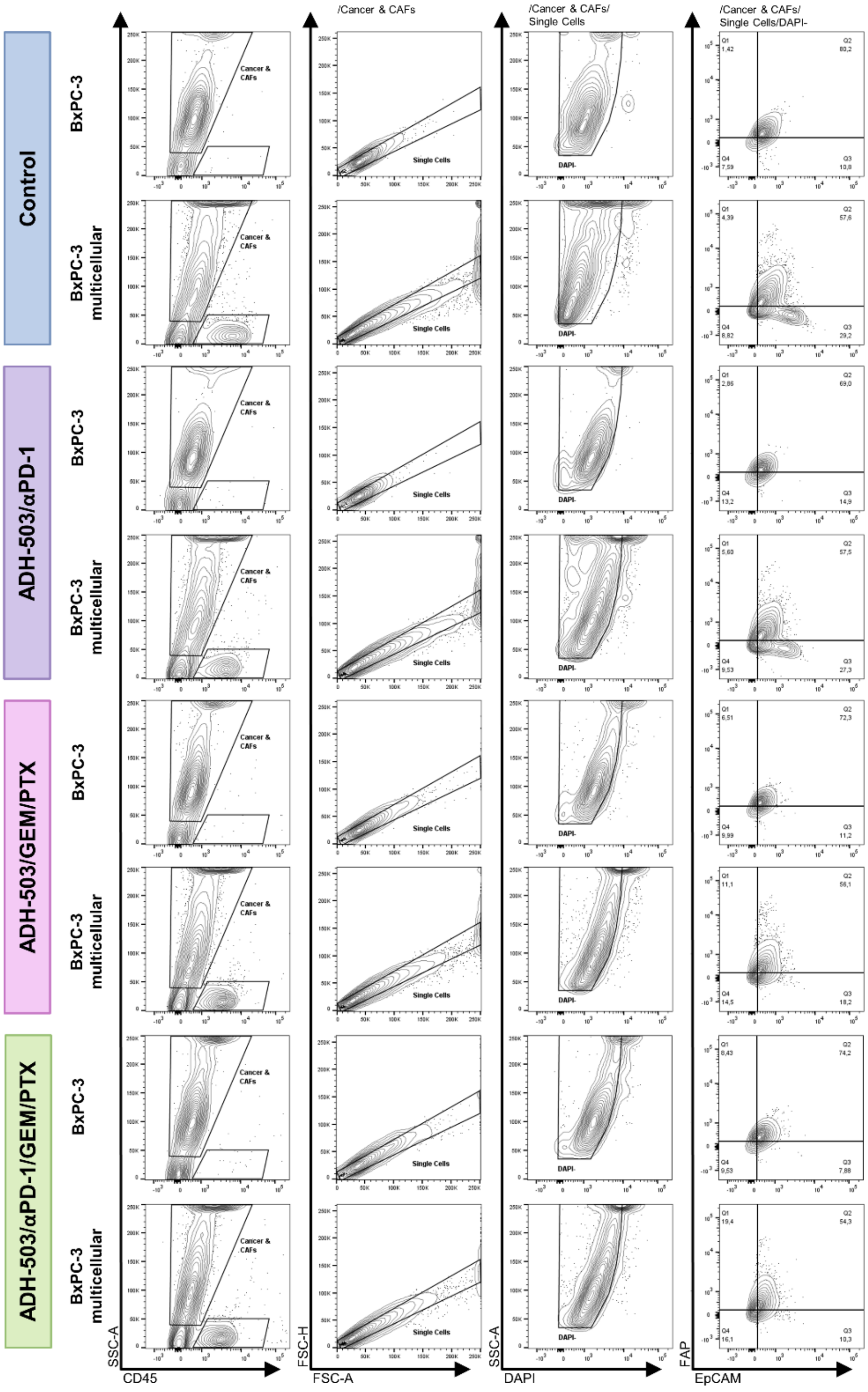

Supplementary Figure S6. Gating strategy for BxPC-3 cells. Gating strategy to separate total PBMCs from CAFs and PDAC cells via CD45 expression, forward scatter characteristics (single cells), and exclusion of a viability dye (DAPI$^-$). A neutral density filter (ND1.0) was incorporated in flow cytometry to enable the analysis of multicellular 3D cultures. n=4

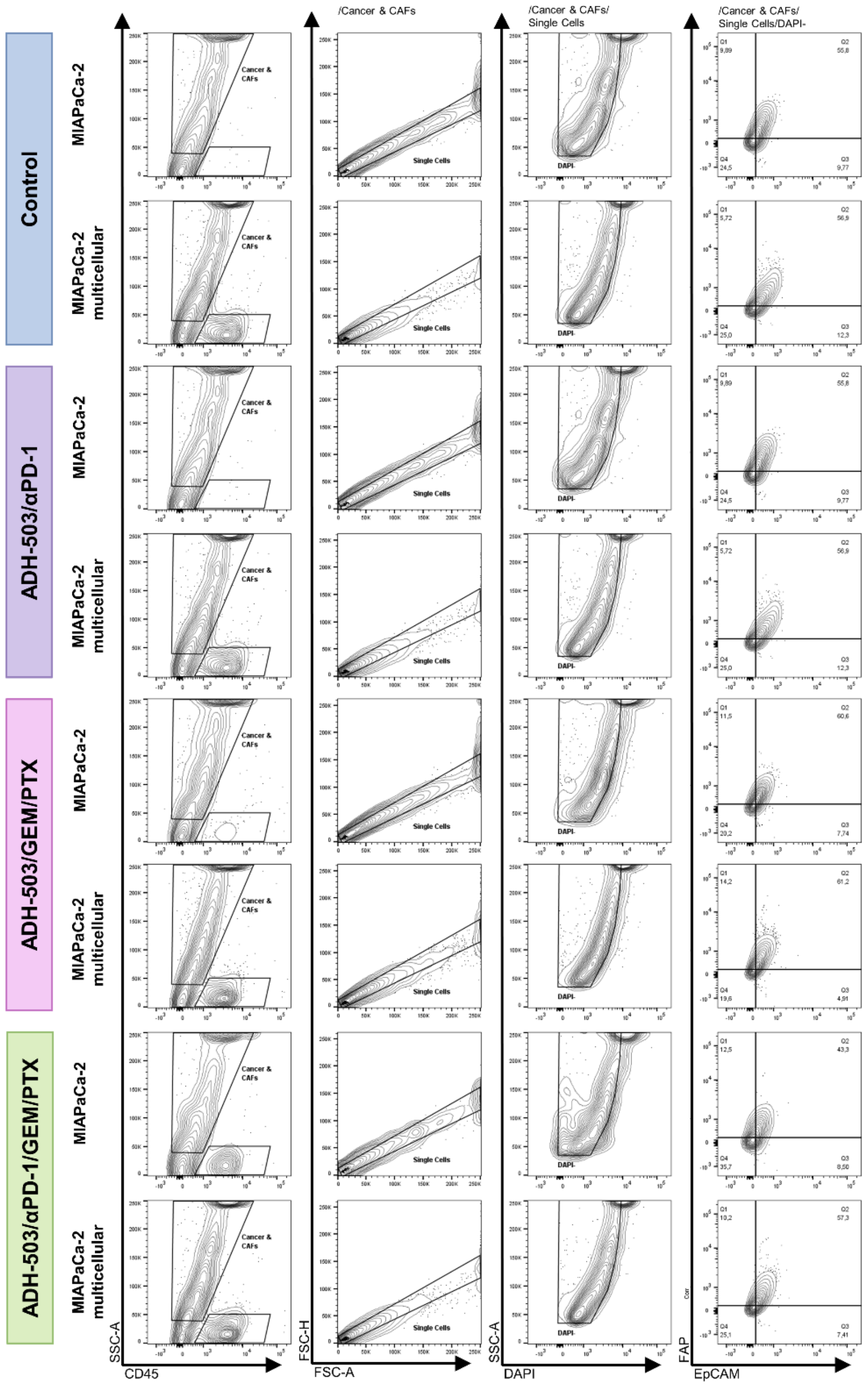

Supplementary Figure S7. Gating strategy for MIAPaCa-2 cells. Gating strategy for a treated sample to separate total PBMCs from CAFs and PDAC cells via CD45 expression, forward scatter characteristics (single cells), and exclusion of a viability dye (DAPI^{-}). A neutral density filter (ND1.0) was incorporated in flow cytometry to enable the analysis of multicellular 3D cultures. n=4

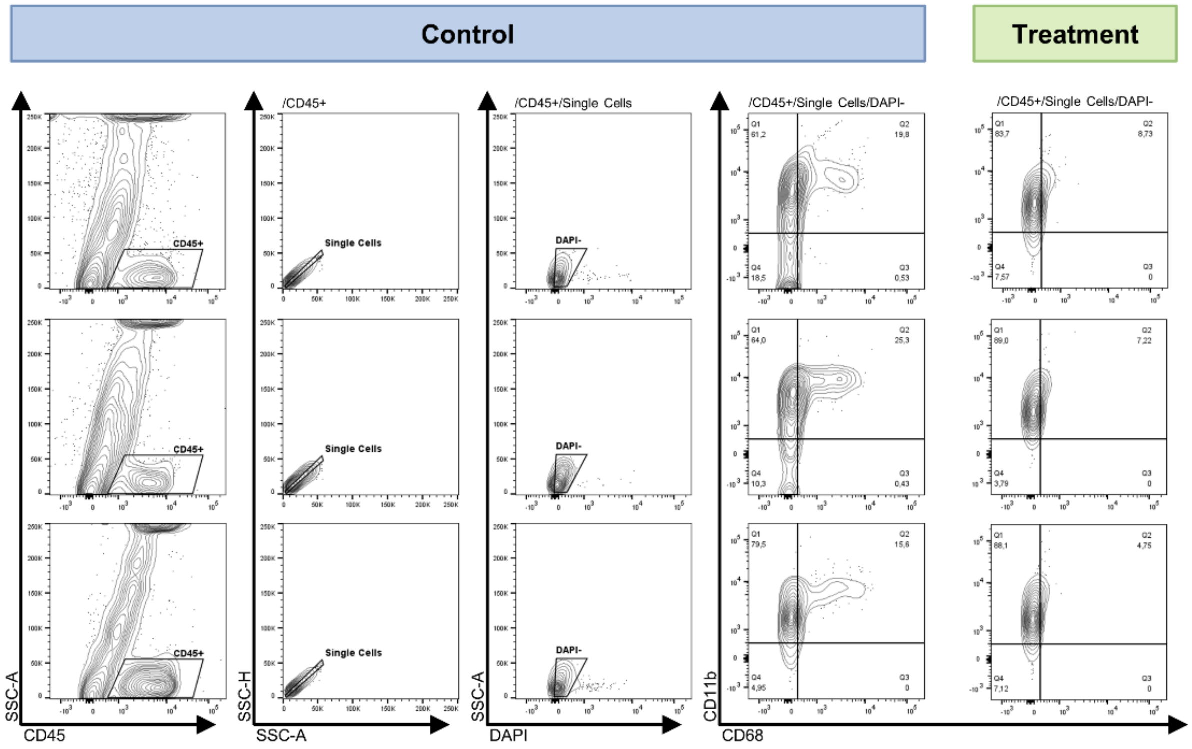

Supplementary Figure S8. Gating strategy for stromal cells. Gating strategy of treated samples to separate total PBMCs from CAFs and PDAC cells via CD45 expression, side scatter characteristics (single cells), and exclusion of a viability dye (DAPI$^-$). A neutral density filter (ND1.0) was incorporated in flow cytometry to enable the analysis of multicellular 3D cultures. n=4

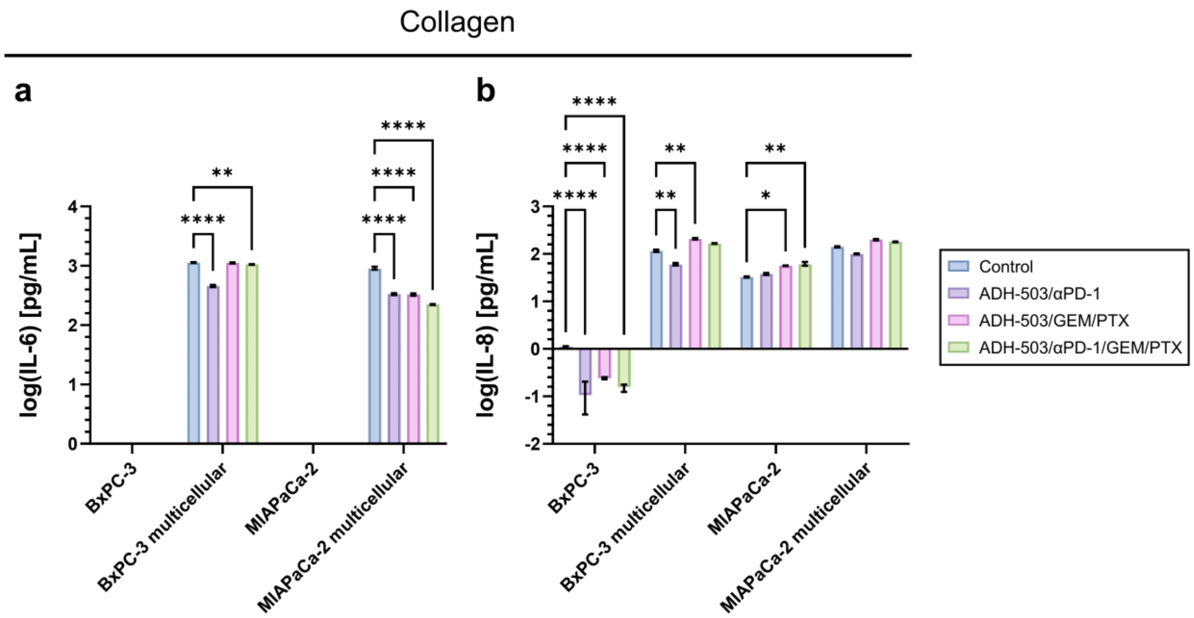

Supplementary Figure S9. Change in cytokine secretion of cancer cell cultures in collagen gels. a, Change in IL-6 secretion in PDAC mono- and multicellular 3D cultures after 4 days of treatment. **b**, Modification of IL-8 expression in PDAC mono- and multicellular 3D cultures after 4 days of treatment. n=3, *$p \leq 0.05$, **$p \leq 0.01$, ***$p \leq 0.001$, ****$p \leq 0.0001$.

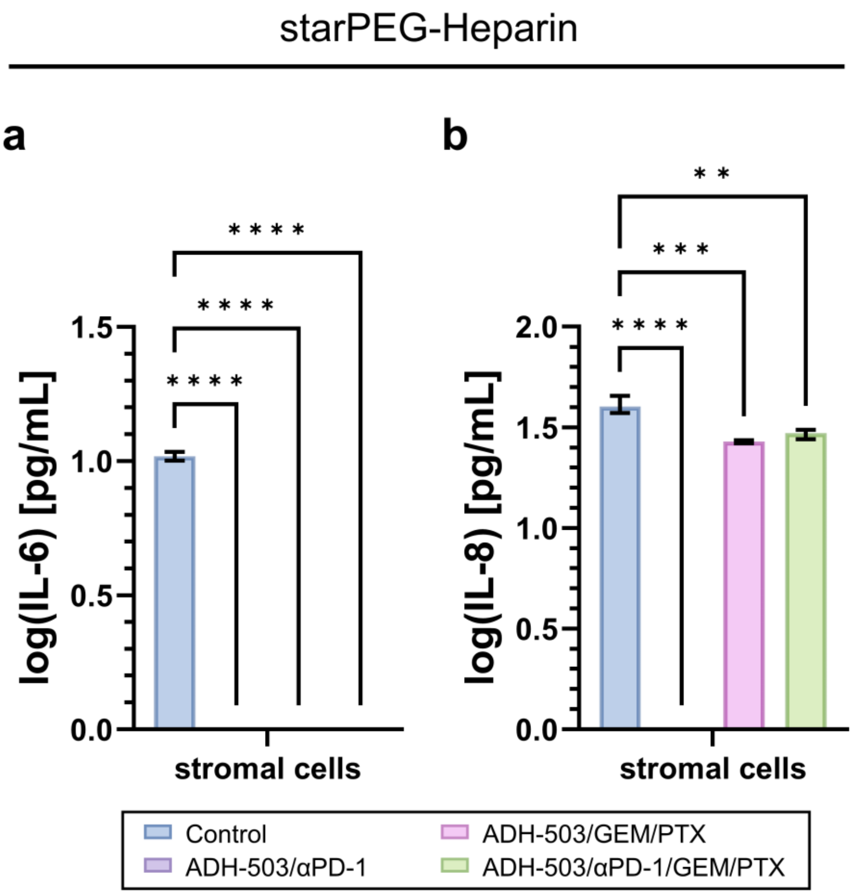

Supplementary Figure S10. Change in cytokine secretion of stromal cell cultures in starPEG-heparin hydrogels. a, Change in IL-6 secretion in stromal cell cultures after 7 days of treatment. **b**, Modification of IL-8 expression in stromal cell cultures after 7 days of treatment. n=3, **$p \leq 0.01$, ***$p \leq 0.001$, ****$p \leq 0.0001$.

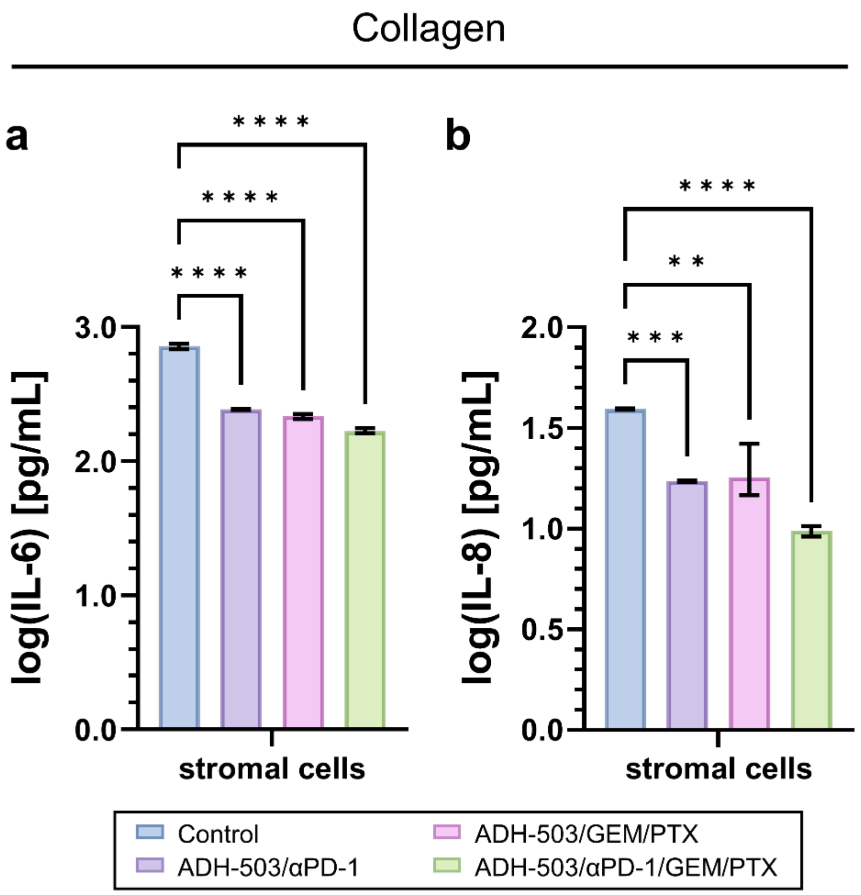

Supplementary Figure S11. Change in cytokine secretion of stromal cell cultures in collagen gels. a, Change in IL-6 secretion in stromal cell cultures after 4 days of treatment. **b**, Modification of IL-8 expression in stromal cell cultures after 4 days of treatment. n=3, $^{**}p \leq 0.01$, $^{***}p \leq 0.001$, $^{****}p \leq 0.0001$.

Supplementary Table S1. Antibodies used for flow cytometry (FC) and immunocytochemistry (ICC)

Antibody	Company	Catalog number	FC/ICC	Dilution
α-human CD11b, PE	Biolegend	982606, # ICRF44	FC	1:100
α-human CD45, Brilliant Violet 785™	Biolegend	304047, # HI30	FC	1:100
α-human CD279 (PD-1), APC/Cyanine7	Biolegend	329922, #EH12.2H7	FC	1:50
α-human CD326 (EpCAM), Brilliant Violet 650™	Biolegend	324225, #9C4	FC	1:100
α-human CD68, APC	Biolegend	333809, #Y1/82A	FC	1:100
α-human alpha-FAB, APC	R&D Systems	FAB3715A, #427819	FC	1:100
α-SMA	Abcam	ab124964, #EPR5368	ICC	1:250
α-CD68	Abcam	ab955, #KP1	ICC	1:100
α-Ki671	Abcam	ab15580	ICC	1:1000
α-cleaved caspase-3	Cell Signaling	9664, #5A1E	ICC	1:1000
Alexa488 α-rabbit	Invitrogen	A-11008	ICC	1:200
Alexa568 α-mouse	Invitrogen	A-11004	ICC	1:200

Supplementary Table S2. Comparison between PDAC PBMCs with the healthy donor before encapsulation

Parameter	PDAC PBMCs	Healthy donor PBMCs
$CD45^+$ (of all events)	68.40%	93.10%
Viable ($DAPI^-$ of $CD45^+$)	84.80%	93.10%
$CD11b^+$ (of $CD45^+$ $DAPI^-$)	75.90%	76.80%
$CD68^+$ (of $CD45^+$ $DAPI^-$ $CD11b^+$)	0.00%	0.07%
$PD\text{-}1^+$ (of $CD45^+$ $DAPI^-$)	27.20%	20.90%
$EpCAM^+$ (of $CD45^+$ $DAPI^-$)	0.04%	0.11%
